# Expression and mutagenesis studies in the *Medicago truncatula* iron transporter MtVTL8 confirm its role in symbiotic nitrogen fixation and reveal amino acids essential for transport

**DOI:** 10.3389/fpls.2023.1306491

**Published:** 2024-01-04

**Authors:** Jingya Cai, Antonella Longo, Rebecca Dickstein

**Affiliations:** Department of Biological Sciences and BioDiscovery Institute, University of North Texas, Denton, TX, United States

**Keywords:** *Medicago truncatula*, symbiotic nitrogen fixation, iron transporter, site-directed mutagenesis, protein models, transporter function

## Abstract

The model legume *Medicago truncatula* establishes a symbiosis with soil bacteria (rhizobia) that carry out symbiotic nitrogen fixation (SNF) in plant root nodules. SNF requires the exchange of nutrients between the plant and rhizobia in the nodule that occurs across a plant-derived symbiosome membrane. One iron transporter, belonging to the Vacuolar iron Transporter-Like (VTL) family, MtVTL8, has been identified as essential for bacteria survival and therefore SNF. In this work we investigated the spatial expression of *MtVTL8* in nodules and addressed whether it could be functionally interchangeable with a similar nodule-expressed iron transporter, MtVTL4. Using a structural model for MtVTL8 and the previously hypothesized mechanism for iron transport in a phylogenetically-related Vacuolar Iron Transporter (VIT), EgVIT1 with known crystal structure, we identified critical amino acids and obtained their mutants. Mutants were tested *in planta* for complementation of an SNF defective line and in an iron sensitive mutant yeast strain. An extended phylogenetic assessment of VTLs and VITs showed that amino acids critical for function are conserved differently in VTLs vs. VITs. Our studies showed that some amino acids are essential for iron transport leading us to suggest a model for MtVTL8 function, one that is different for other iron transporters (VITs) studied so far. This study extends the understanding of iron transport mechanisms in VTLs as well as those used in SNF.

## Introduction

Symbiotic nitrogen fixation (SNF) in legumes uses energy derived from photosynthesis to reduce N_2_ gas to ammonia at normal temperature and pressure, and is thus, especially important for sustainable food production. Legumes form a symbiosis with bacteria called rhizobia, which are hosted in specialized root organs called nodules (Hirsch et al., 1992; Oldroyd et al., 2011). These form after a series of interactions between the legume root cells and rhizobia which results in the reprogramming and division of plant cortical cells to form nodule primordia. These primordial cells are colonized by rhizobia in infection threads that deliver the rhizobia to the cells (Oldroyd, 2013; Roy et al., 2020). Rhizobia enter the plant cells in a process resembling endocytosis in which rhizobia are surrounded by a plant-derived symbiosome membrane (SM), through which all plant-microbe nutrient exchange occurs (Udvardi and Poole, 2013). The internalized rhizobia surrounded by the SM and the symbiosome space form symbiosomes, novel organelle-like structures. Rhizobia within symbiosomes, now called bacteroids, grow and divide until tens of thousands fill the infected cells, which increase in ploidy depending on the legume species (Maróti and Kondorosi, 2014). The outer cells of nodules form a gas diffusion barrier resulting in a hypoxic nodule interior (Witty et al., 1987) protecting the rhizobially-encoded oxygen-labile nitrogenase enzyme. Paradoxically, high rates of respiration in nodules are required by rhizobia as well as plant mitochondria during SNF. Respiration is supported by the rapid binding and delivery of oxygen by leghemoglobin, lowering oxygen concentrations further in the nodule interior (Appleby, 1984; Ott et al., 2005).

Many legumes, like the model plant *Lotus japonicus* and soybean, form round, determinate nodules that only have a transient meristem. Others, like the model *Medicago truncatula* and pea, form oblong, indeterminate nodules with a persistent meristem. Both nodule types have a central zone of rhizobia infected cells interspersed with uninfected cells (Hirsch, 1992). Indeterminate nodules contain cells in a developmental gradient classified into zones. Zone I (ZI) is the meristem; zone II (ZII) is the invasion zone where rhizobia enter plant cells, divide and differentiate; the interzone (IZ) is where final maturation occurs; and zone III (ZIII) is where SNF takes place. Older nodules also have a senescent zone, zone IV (IV), which likely is involved in nutrient recycling (Vasse et al., 1990). Each zone has characteristic gene expression (Limpens et al., 2013; Roux et al., 2014).

The transition metal iron is a key essential nutrient required for SNF (Clarke et al., 2014; González-Guerrero et al., 2014; Day and Smith, 2021; González-Guerrero et al., 2023) and it accumulates to higher concentrations in nodules than other plant organs (Burton et al., 1998). Low iron levels can hinder SNF in nodules (O'Hara et al., 1988; Tang et al., 1992; Johnston et al., 2001). Iron is a cofactor of multiple metallo-enzymes directly or indirectly implicated in SNF in different stages of the symbiosis process. Rhizobial catalase, containing a catalytic heme iron, has been implicated as having a crucial role early in bacterial infection of nodule primordia (Jamet et al., 2003). Later in nodule development, heme iron is required by rhizobia to sense declining O_2_ levels (Gilles-Gonzalez et al., 1991), signaling the rhizobia to develop into SNF-capable forms. As rhizobia mature to be able to fix nitrogen, they express nitrogenase comprising the iron-sulfur cluster containing NifH and the iron-molybdenum cofactor-containing NifDK (Hoffman et al., 2014; Einsle and Rees, 2020). Proteins essential for shuttling reducing equivalents to nitrogenase, e.g. FixABCX, contain iron cofactors (Ledbetter et al., 2017). Rhizobial proteins essential for respiration contain iron, either as heme iron (Preisig et al., 1996) or as iron-sulfur clusters. In the plant cell cytosol, leghemoglobin containing heme iron, is abundant, buffering and transporting oxygen to the respiring rhizobia and mitochondria (Appleby, 1984; Ott et al., 2005).

Evidence suggests that iron reaches the nodule via the xylem, chelated to citrate or to nicotianamine, where it is released from the vasculature into the apoplastic space in ZII, the infection and differentiation nodule zone (Rodríguez-Haas et al., 2013). The route from vasculature to infected cells crosses several cell layers, which the iron traverses using symplastic and apoplastic routes (Brear et al., 2013; Rodríguez-Haas et al., 2013; Day and Smith, 2021; González-Guerrero et al., 2023). *M. truncatula* NRAMP1, a member of the Natural Resistance-Associated Macrophage Protein (NRAMP) family, transports iron from the apoplast into the infected cells’ cytosol (Tejada-Jiménez et al., 2015), potentially assisted by citrate efflux transporter MtMATE67 (Multidrug And Toxic Compound Extrusion) (Kryvoruchko et al., 2018). MtMATE67 transports citrate in an iron-activated manner and appears to enhance iron uptake into infected cells (Kryvoruchko et al., 2018). Once inside the cytoplasm of nodule cells, the iron needs to cross another membrane, the SM, to reach the internalized rhizobia. In *M. truncatula*, two different transporters have been implicated in the SM transport: MtVTL8 (*M. truncatula* Vacuolar Iron Transporter (VIT)-Like) (Walton et al., 2020; Cai et al., 2022), the subject of this study, and MtFPN2 (*M. truncatula* Ferroportin Protein2) (Escudero et al., 2020). Both MtVTL8 and MtFPN2 are ferrous iron efflux transporters. Plants with mutations in either protein’s gene show defects in SNF (Escudero et al., 2020; Walton et al., 2020; Cai et al., 2022).

Rhizobia inside symbiosomes import iron from the symbiosome space. In rhizobia, iron uptake is regulated by the heme-regulated transcription repressor Irr, that represses *rirA.* RirA is a transcriptional activator of iron-uptake genes (O'Brian, 2015; Sankari et al., 2022). The nodule cysteine-rich peptide (NCR) NCR247 is secreted into symbiosomes and taken up by rhizobia in a *bacA*-dependent manner (Marlow et al., 2009; Farkas et al., 2014). NCR247 was recently found to bind to and sequester heme, inducing an iron starvation response in rhizobia, resulting in increased iron import (Sankari et al., 2022). Thus, NCR247 enables rhizobia to be better iron sinks.

MtVTL8 is a member of the Cross-Complements Ca^2+^ phenotype of the csg1/Vacuolar Iron Transporter (CCC1/VIT) family. Both it (encoded by Medtr4094335/MtrunA17Chr4g0050851) and the homolog MtVTL4 (encoded by Medtr4g09325/MtrunA17Chr4g0050811) are expressed in nodules, although their expression profiles are somewhat different temporally and spatially (Roux et al., 2014; Walton et al., 2020; Carrere et al., 2021). MtVTL8 was found to localize on the SM; while MtVTL4 localizes to the plasma membrane and membranes surrounding the infection thread (Walton et al., 2020). Plants with mutations in *MtVTL8* have profound defects in nodule development, while those with mutations in *MtVTL4* have only minor defects (Walton et al., 2020; Cai et al., 2022). The two *Tnt1 mtvtl4* mutants studied were not back-crossed and had similar vegetative growth defects in both low and high nitrogen conditions, with higher numbers of less-developed nodules, suggesting a developmental defect, not a symbiotic defect *per se* (Walton et al., 2020). *MtVTL8* is homologous to *L. japonicus LjSEN1* (Stationary Endosymbiont Nodule 1) and to soybean *GmVTL1a*, which when knocked down or mutated have similar phenotypes to *mtvtl8* mutants (Suganuma et al., 2003; Hakoyama et al., 2012; Brear et al., 2020; Liu et al., 2020). *MtVTL4*, *MtVTL8* and *GmVTL1a* are able to complement the yeast *Saccharomyces cerevisiae Δccc1* (*ScΔccc1*) mutant with a defect in a vacuolar ferrous iron transporter demonstrating their iron efflux activity (Brear et al., 2020; Walton et al., 2020). Two *mtvtl8* mutants are available. The first is *mtvtl8-1* or 13U, in the A17 genotype, having a large deletion on chromosome 4 that deletes both *MtVTL4* and *MtVLT8* (Walton et al., 2020). The second is *mtvtl8-2* derived from *Tnt1* line NF11322 in the R108 background with a homozygous *Tnt1* insertion in the exon of *MtVTL8* (Cai et al., 2022). Both *mtvtl8-1 and mtvtl8-2* form defective Fix^-^ white nodules with *Sinorhizobium* (*Ensifer*) *meliloti* (hereafter referred to as *S. meliloti*) under limited nitrogen conditions (Walton et al., 2020; Cai et al., 2022).

VIT transporters are predicted to fold into five transmembrane helices (TMH) with a long hydrophilic sequence between TMH2 and TMH3. Similarly, the related VTL transporters contain five TMHs but have a much shorter sequence between TMH2-3. Iron transporters with truncated sequences or VTLs are found among angiosperm plants, both monocots and eudicots (Sorribes-Dauden et al., 2020). Based on phylogenetic analysis of CCC1/VTL sequences, Sorribes-Dauden et al. (2020) proposed a different origin for VITs and VTLs: VITs originated from an ancestral horizontal transfer from bacteria while VTLs were transferred from an archaeal lineage with both transfers dating at the origin of the last common eukaryote ancestor.

The crystal structure of VIT1 from the rose gum *Eucalyptus grandis* was solved (Kato et al., 2019) and is so far the only solved iron transporter structure from the CCC1/VIT family. The structure confirmed the presence of five TMHs, with the N-terminal end located in the cytoplasm and the C-terminal end in the vacuolar space. In the structure, EgVIT1 is a dimer with TMH1 from each monomer at the center and TMH2-5 arranged clockwise around TMH1 to form the transmembrane domain (TMD). A cavity forms between the monomers and in the crystal structure is open toward the cytoplasm. The cytoplasmic TMH2-3 loop folds into three short α-helices, named H1, H2, and H3. Glutamic residues from H1 and H3 in combination with two glutamic acids from the extended cytoplasmic portion of TMH2 bind three metal ions in each monomer. This region was therefore defined as a metal binding domain (MBD). The dimeric interaction is mediated both by the TMD and the MBD. EgVIT1 as well as another member of the CCC1/VIT family, PfVIT, from the human malaria-causing parasite *Plasmodium falciparum*, were shown to be Fe^2+^/H^+^ antiporters with the exchange driven by the proton electrochemical potential (Labarbuta et al., 2017; Kato et al., 2019). Comparing the EgVIT1 structure with the VTL sequences suggests the absence of an MBD in the subfamily (Sorribes-Dauden et al., 2020).

In this work, we investigated the spatial expression of *MtVTL8* in WT and *mtvtl8-2* roots during nodulation. We then explored whether altering *MtVTL4*’s expression using the *MtVTL8 cis* elements would enable it to functionally complement *mtvtl8-2* roots. We used a comparison between the EgVIT1 structure to a structural model of MtVTL8 to identify potentially essential amino acids. We then performed mutagenesis studies to challenge our hypothesis. Our results confirm MtVTL8’s unique role as an iron transporter in nodulation and suggest that it may function differently from previously characterized iron transporters. Models for potential mechanisms of transport are presented.

## Materials and methods

### MtVTL8 structural model

A structural model for MtVTL8 was obtained from the AlphaFold protein structure database (Jumper et al., 2021; Varadi et al., 2022) using MtVTL8’s UniProt ID: A0A072UNI3. Structural analysis was limited to residues 40-235 due to the low confidence score for the N-terminal amino acids. Structures were visualized by the PyMOL Molecular Graphics System (Schrödinger, LLC). A dimer was generated within PyMOL by aligning the AlphaFold monomer with chains A and B of EgVIT1, pdb 6IU3 (Kato et al., 2019). Structures were positioned in the lipid membrane using the PPM web server (Lomize et al., 2012).

### Primers and plasmids

Primers and plasmids used for this study are listed in **Supplementary Tables S1**, **S2**, respectively. Vectors pMU06 and pMU14 were generous gifts of Drs. Wei Liu and Michael Udvardi. pMU06 contains the *pAtUBI-DsRed* marker gene for detection of transformed roots using DsRed fluorescence, a site to insert a promoter upstream of *GUS*, the *GUS* gene, and the *35S 3’* terminator. pMU14 contains the *pAtUBI-DsRed* marker gene, the *35S* promoter, *GFP* and *35S 3’* terminator. All constructs were confirmed by sequencing.

### Cloning *MtVTL8* and *MtVTL4* for expression in *M. truncatula*

For *in planta* complementation studies, *MtVTL*s were cloned in the binary vector pMU14 for expression in Medicago as in our previous study of the nitrate transporter MtNPF1.7 (Yu et al., 2021). To express *MtVTL8* with its native controlling elements the *MtVTL8* promoter (2629 bp), *MtVTL8* CDS (708 bp) and *MtVTL8* terminator (1083 bp) were amplified from *M. truncatula* R108 genomic DNA using Q5 High-Fidelity DNA polymerase (New England Biolabs) and primers JYC05-F1/JYC05-R1, JYC05-F2/JYC05-R2, and JYC05-F3/JYC05-R3 respectively. pMU14 was digested with *Xho*I and *Hin*dIII-HF (New England Biolabs). The digested vector, the *MtVTL8* promoter, CDS, and terminator PCR fragments were assembled with the Gibson assembly method (NEBuilder HiFi DNA Assembly, New England Biolabs) to form pJYC05 (pMU14/p*MtVTL8*-Mt*VTL8*-*MtVTL8-t*). To obtain the *MtVTL4* gene driven by p*MtVTL8*, *MtVTL4* was amplified with forward primer JYC24F and reverse primer JYC24R from R108 gDNA. It was assembled with the pJYC05-*Spe*I/*Kpn*I fragment with the Gibson assembly method to form pJYC24 (pMU14/p*MtVTL8-MtVTL4*-*MtVTL8-t*). Plasmids were transformed into electrocompetent *Agrobacterium rhizogenes* MSU440 by electroporation using Gene Pulser Xcell™ electroporation system (Bio-rad, Hercules, CA, USA) (Valimehr et al., 2014).

### Construct for p*MtVTL8-GUS* expression

The *GUS* CDS was amplified from the binary vector pMU06 by using primers JYC15-F and JYC15-R. The *GUS* CDS and JYC05-*Spe*I-*Kpn*I were assembled with the Gibson assembly method (NEBuilder HiFi DNA Assembly, New England Biolabs) to form pJYC15 (MU06/p*MtVTL8-GUS*). The construct was introduced into *A. rhizogenes* as above.

### GUS staining and nodule sections

Plant roots transformed with p*MtVTL8-GUS* were selected by their red fluorescence, demonstrating presence of the transformed vector with the visible marker, at 0, 5 and 15 dpi. Transformed roots were harvested in 0.1 M PBS (Na_2_HPO_4_ and NaH_2_PO_4_, pH 7.0) and then transferred to GUS solution (44.5 mL of 100 mM PBS-Na pH 7.0, 2 mL of 50 mM K_3_Fe(CN)_6_, 2 mL of 50 mM K_4_Fe(CN)_6_, 1 mL of 0.5 M EDTA, 0.5 mL of 10% Triton X-100, 50 mg of X-Gluc salt mixed together) followed by vacuum infiltration for 1.5 h. Roots were kept at 37**°**C overnight. The samples were subsequently washed with 0.1 M PBS (Na_2_HPO_4_ and NaH_2_PO_4_, pH 7.0) at 4**°**C. Samples were observed under an Olympus BX50 microscope (Olympus, Tokyo, Japan). GUS-stained nodules were cut and fixed with 4% glutaraldehyde (made in 0.1 M PBS-Na, pH 7.0) under vacuum for 30 min. The samples were kept overnight at 4**°**C with gentle rotation. Then, the samples were washed three times with 0.1 M PBS-Na and dehydrated with a series of ethanol gradients (30%, 50%, 70%, 90%, 100%), each step was carried out with gentle rotation for 30 min at room temperature. The ethanol was replaced with ethanol: Technovit 7100 (Kulzer Technik, Hanau, Germany) (2:1/v:v) and rotated for one hour at room temperature, followed by ethanol: Technovit 7100 (1:2/v:v) for another hour. The liquid was replaced with 100% Technovit 7100 and rotated gently at room temperature overnight. The next day, the liquid was replaced with fresh Technovit 7100 and rotated at room temperature for 1 h. Resin was prepared by mixing Technovit 7100 with hardener II (15:1/v:v). Resin was added to the mold well and the nodule was placed in the resin. The well was covered with parafilm and left at room temperature for 1 h for polymerization. After polymerization, the parafilm was removed and the block was glued to an adaptor using the Technovit 3040 glue (Kulzer Technik, Hanau, Germany). Five micrometer nodule sections were sliced by a microtome (Leica HistoCore Multicut, Leica) and collected on glass slides. The slides were stained with ruthenium red staining solution (200 mg ruthenium red, 200 mL water) for 5 min followed by rinsing with water until the background was clear. The slides were dried on a hotplate and nodule sections were visualized with an Olympus BX50 microscope.

### Mutagenesis of *MtVTL8* for expression in *M. truncatula*

Mutants of *MtVTL8* were constructed in pJYC05, replacing the *MtVTL8* cDNA with the mutated gene. Two PCR reactions were carried out resulting in two overlapping fragments, one containing the 5’ end of *MtVTL8* to the desired mutation (using primers JYCmut-F and JYC19R2) and the other containing the desired mutation to the 3’ end of *MtVTL8* (using primers JYC19F2 and JYCmut-R). The relevant primers for each construct are listed in **Supplementary Table S3**. Mutated *MtVTL8* fragments were subsequently assembled into pJYC05-*Spe*I/*Kpn*I vector with the Gibson assembly method to form pJYCmut. We obtained the following mutants: MtVTL8_R51A (pJYC16), MtVTL8_D59A (pJYC17), MtVTL8_G88E (pJYC18), MtVTL8_E100A (pJYC19), MtVTL8_E111A (pJYC20), and MtVTL8_K135A (pJYC21). Double mutants MtVTL8_R51E/E100R (pJYC22), and MtVTL8_E111K/K135E (pJYC23) were obtained by the assembly of three PCR fragments with pJYC05-*Spe*I/*Kpn*I vector.

### Complementation experiments in *mtvtl8-2* plants

*M. truncatula Tnt1* insertion mutant *mtvtl8-2* seeds and control wild type R108 seeds were scarified and germinated as described (Cai et al., 2023). Seedlings of *mtvtl8-2* and R108 were transformed with *A. rhizogenes* MSU440 containing either empty vector (EV) pMU14, pJYC05, or plasmids containing mutated *MtVTL8*. Transformants were transferred to Fåhraeus medium (Fåhraeus, 1957) containing 5 mg/L nystatin (Millipore-Sigma, Burlington, MA USA) for 5 d in growth chamber with 14 h/10 h (light/dark) at 24°C (Boisson-Dernier et al., 2001). Then, the plants were moved to a 1% MS medium (Millipore-Sigma) plate in between two filter papers covering the roots for 14 d in growth chamber with 14 h/10h (light/dark) at 24°C. Transformed roots were selected based on their expression of the DsRed fluorescent marker, contained in the pMU14 vector. This was done using a Leica MZ10F dissecting microscope (Leica, Deer Park, IL, USA). Non-transformed roots were excised. The plants with transgenic roots were transferred to the aeroponic system with Lullien’s medium (Lullien et al., 1987) without a nitrogen source at 22°C with a 16 h/8 h light/dark photoperiod at 60 μmol m^-2^s^-1^ for 5 d (Barker et al., 2006; Cai et al., 2023). Then, the plants were inoculated with *S. meliloti* Rm41 (Kondorosi et al., 1980). Growth of the transformed plants was checked at 15 dpi and nodules were analyzed and documented using the Leica MZ10F dissecting microscope. Nodules were photographed and their proxy 2-D surface areas were analyzed with Fiji software (Schindelin et al., 2012). For chlorophyll estimation, we followed the control method described (Liang et al., 2017). Briefly, leaves were collected at 28 dpi and frozen in liquid N_2_. For extraction, 100 mg of leaves were ground under liquid N_2_, 1.0 mL of 80% acetone was added and the mixture gently agitated for 24 h at room temperature, followed by 15,000*g* centrifugation for 5 min at 4°C. 100 μL of supernatant was mixed with 400 μL of 80% acetone. Absorbance of the supernatant was measured at wavelength of 645 nm (A645) and 663 nm (A663) with a Bio-Rad SmartSpec Plus spectrophotometer (Hercules, CA, USA) The total chlorophyll content was calculated following the Arnon’s equation (Arnon, 1949): Total chlorophyll (μg/mL) = 20.2 (A645) + 8.02 (A663).

### Cloning for yeast expression

The vector pYES2/CT (ThermoFisher Scientific) was digested with HindIII-HF and BamHI-HF (New England Biolabs). The *MtVTL8* gene and its eight different mutant versions were amplified from pJYC16-23 using primers JYC10.FOR and JYC10.REV and assembled with the pYES2/CT-HindIII/BamH1 fragment using the Gibson assembly method to form pJYC10 (*MtVTL8-wt*) and eight different *Mtvtl8-mut* versions (pYJYC16-23).

### Fe^2+^ sensitivity in yeast

The *Saccharomyces cerevisiae* wild type strain DY150 and mutant strain Δ*ccc1*(*ura3, leu2, his3, ade2, can1, CCC1::HIS3*) (Li et al., 2001) in the DY150 background were grown in YPD medium at 30°C, 250 rpm for 16 h. Competent cells of DY150 and Δ*ccc1* were produced using the Frozen-EZ Yeast Transformation II kit (Zymo Research). pYES2/CT was transformed into the DY150. The mutant strain Δ*ccc1* was transformed with pYES2/CT, pYES2-*AtVTL1* (At1g21140, wild type), pJYC10 or pYJYC16-23. Single colonies, each with a specific construct, were cultured in SC-U+Gal medium at 28°C for 16* h*. OD_600_ was monitored and adjusted to 1 for the spot assay. Ten μL of each culture and their serial dilutions were spotted on SC-U+Gal or SC-U+Gal + 5 mM ammonium ferrous sulfate plates, followed by incubation at 28°C for 3 d. Plates were observed to assess the yeast growth and photographed.

### Sequence analysis and phylogenetic tree construction

Genes belonging to the *CCC1/VIT1* family were identified in the PANTHER18 family library of trees (Thomas et al., 2022) using the keyword PTHR31851. Sequences from one alga and selected plant genomes were retrieved from UniProt Knowledgebase (UniProtKB) (UniProt Consortium, 2023). Sequences for *Ceratopteris richardii* and *Lotus japonicus* were obtained on Phytozome v13 (Goodstein et al., 2012). We limited our analysis to the following genomes: *Chlamydomonas reinhardtii* (green alga); *Selaginella moellendorffii* (lycophyte); *Marchantia polymorpha* (liverwort); *Physcomitrella patens* (bryophyte); *Amborella trichopoda* (amborella); *Ceratopteris richardii* (pteridophyte); *Brachypodium distachyon*, *Hordeum vulgare*, *Musa acuminata*, *Oryza sativa*, *Setaria italica*, *Sorghum bicolor*, *Triticum aestivum*, *Tulipa gesneriana*, *Zea mays*, and *Zostera marina* (monocots)*; Arabidopsis thaliana*, *Brassica napus*, *Capsicum annuum*, *Citrus sinensis*, *Cucumis sativus*, *Erythrante guttata*, *Eucalyptus grandis*, *Glycine max*, *Gossypium hirsutum*, *Helianthus annuus, Lactuca sativa, Lotus japonicus*, *Medicago truncatula*, *Nicotiana tabacum*, *Populus trichocarpa*, *Prunus persica*, *Ricinus communis*, *Solanum lycopersicum, Solanum tuberosum*, *Spinacia oleracea*, *Theobroma cacao*, and *Vitis vinifera* (eudicots). Sequences are listed in **Supplementary Table S4**. Multiple sequence alignment was obtained using ClustalW. WebLogo 3 was used to create the sequence logos (Crooks et al., 2014). The initial maximum-likelihood phylogenetic tree was calculated by ModelFinder (Kalyaanamoorthy et al., 2017) using the IQ-TREE multicore version 2.1.2 COVID-edition for Linux 64-bit. The best-fit model based on Bayesian Information Criterion was JTT+R6. Branch support for the maximum-likelihood tree was generated with ultrafast bootstrap (Hoang et al., 2018) (1000 replicates) implemented in the IQ-TREE software (Nguyen et al., 2015) Calculations were performed on the CIPRES (CyberInfrastructure for Phylogenetic Research) science gateway platform (Miller et al., 2010). The phylogenetic tree was visualized and annotated with iTOL (Letunic and Bork, 2021) and can be accessed here: https://itol.embl.de/tree/4718713120329291698508806. Predicted protein structures, provided by AlphaFold within UniProtKB, were visually analyzed for each protein in the tree.

## Results

### Spatial expression of *MtVTL8* in *M. truncatula*

**Figure 1:**
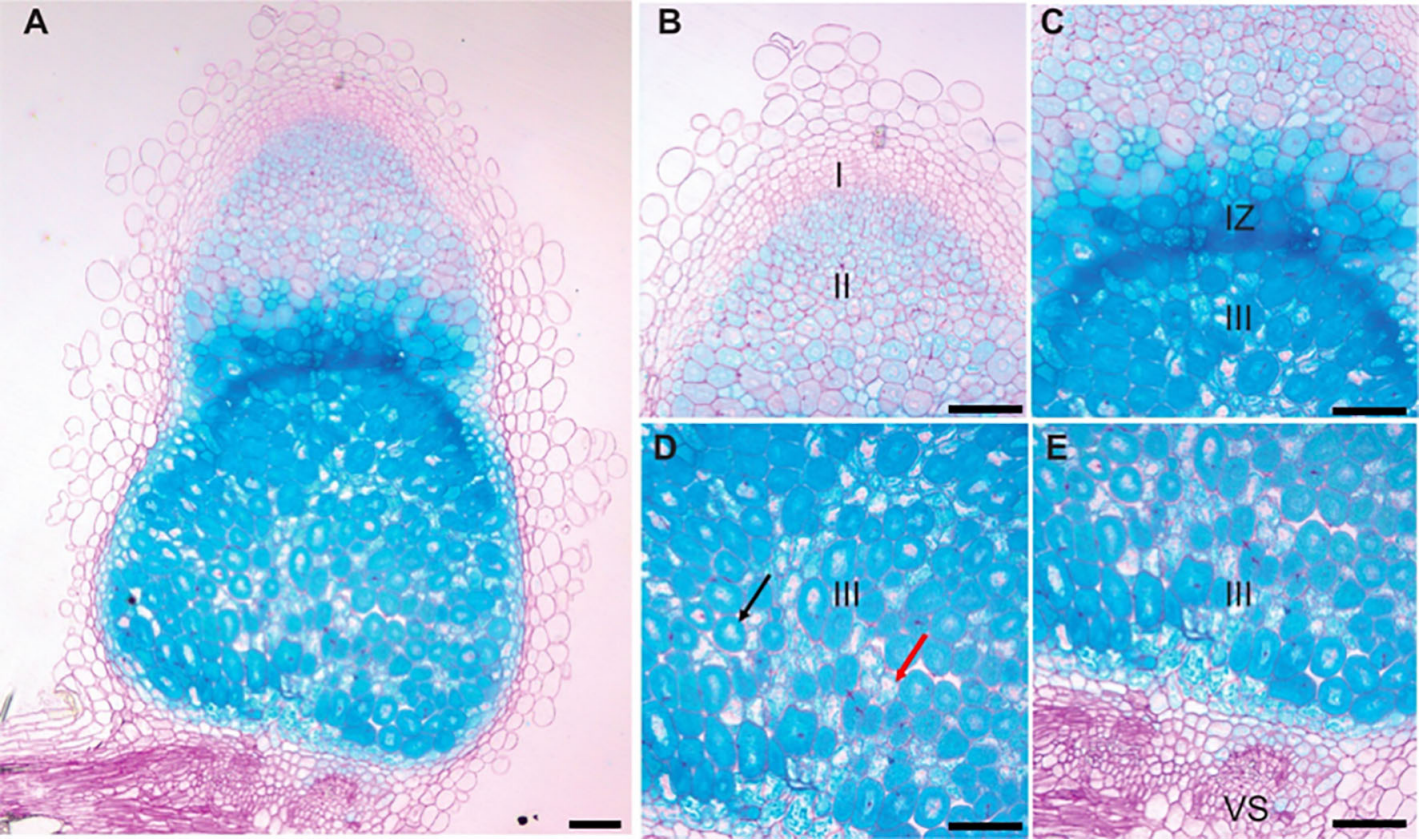
p*MtVTL8-GUS* is expressed from zone II to zone III in both infected and uninfected cells of *Medicago truncatula* genotype R108 nodules. Nodules were collected and stained for GUS followed by fixation with glutaraldehyde. Then they were embedded in Technovit 7100 (Kulzer Technik, Hanau, Germany) and sectioned at 5 μm. **(A)** Expression of p*MtVTL8-GUS* in the R108 nodule section at 15 dpi with *S. meliloti* Rm41. **(B-E)** Details from **(A)** for expression of p*MtVTL8-GUS* from Zone I to Zone III. VS, vasculature. I, zone I, meristem zone. II, zone II, infection zone. IZ, interzone, maturation zone. III, zone III, fixation zone. The black arrow indicates an infected cell. The red arrow indicates an uninfected cell. Scale bars represent 0.1 mm.

To determine when and where *MtVTL8* is expressed in roots and nodules, the coding sequence for the β-glucuronidase (GUS) enzyme (Jefferson et al., 1987) was cloned under the control of the *MtVTL8*-promoter (*pMtVTL8*). The *pMtVTL8*-GUS construct was transferred to an *A. rhizogenes* strain and expressed via hairy-root transformation in the roots of R108 wild type and *mtvtl8-2*, a *Tnt1* line with a homozygous *Tnt1* insertion in the exon of *MtVTL8* (Cai et al., 2022). Roots and nodules were examined at 0 dpi, 5 dpi and 15 dpi with *S. meliloti* Rm41 (**Supplementary Figures S1**, **S2**). Blue color was found in nodule primordia and nodules but not in the roots, demonstrating that the *MtVTL8* promoter highly and exclusively directs expression in these tissues (**Supplementary Figures S1**, **S2**). Longitudinal cross sections of the GUS stained nodules of R108 and *mtvtl8-2* at 15 dpi followed by light microscopy imaging showed that p*MtVTL8-GUS* was expressed from ZII to ZIII in both infected cells (IC) and uninfected cells (UC) with high expression in the infection zone (IZ) (**Figure 1** ; **Supplementary Figure S3**). Expression was not observed in the interzone (IZ). These results correspond to the RNA-seq data from the Symbimics database (Roux et al., 2014) and reinforce the idea that MtVTL8’s role is to support SNF. Expression of p*MtVTL8-GUS* was not observed in the vascular system (**Figures 1A, E**; **Supplementary Figures S3A, B**). In *mtvtl8-2*, the *MtVTL8* expression was dramatically decreased in the premature senescent zone compared to the zones containing rhizobia (**Supplementary Figure S3**). However, the expression pattern in R108 was similar to that observed in *mtvtl8-2*, (**Supplementary Figure S3**), taking into consideration the developmental defects observed in these nodules due to the absence of *MtVTL8* (Walton et al., 2020; Cai et al., 2022). At the 15 dpi time point in R108 nodules, there is not yet a senescent zone; taken together, these results suggest that MtVTL8 may not have a role in reusing Fe^2+^ during senescence.

### Functional complementation of a yeast Fe^2+^ mutant and *mtvtl8-2* nodulated roots

The yeast Cross-complements Ca^2+^ phenotype of the Csg1 family (*CCC1*) (Li et al., 2001) gene encodes an Fe^2+^/Mn^2+^ vacuolar transporter. Yeast *Δccc1* mutants, containing a deletion of *CCC1*, show hypersensitivity to high concentrations of external Fe^2+^ because they fail to sequester the excess Fe^2+^ in their vacuoles (Li et al., 2001). The *ScΔccc1* strain has been used in complementation studies of several plant iron transporters belonging to the CCC1 family including MtVTL4 and MtVTL8 (Walton et al., 2020); AtVIT1 (Kim et al., 2006); AtVTL1, AtVTL2 and AtVTL5 (Gollhofer et al., 2014); TgVIT1 (Momonoi et al., 2009); OsVIT1 and OsVIT2 (Zhang et al., 2012); TaVIT2 (Connorton et al., 2017); EgVIT1 (Kato et al., 2019); GmVTL1a and GmVTL1b (Brear et al., 2020; Liu et al., 2020). Additionally the strain was used to test PfVIT from the *P. falciparum* parasite (Slavic et al., 2016). All tested transporters were able to complement the *ScΔccc1* mutation and restore the growth of the defective strain in high iron conditions.

**Figure 2:**
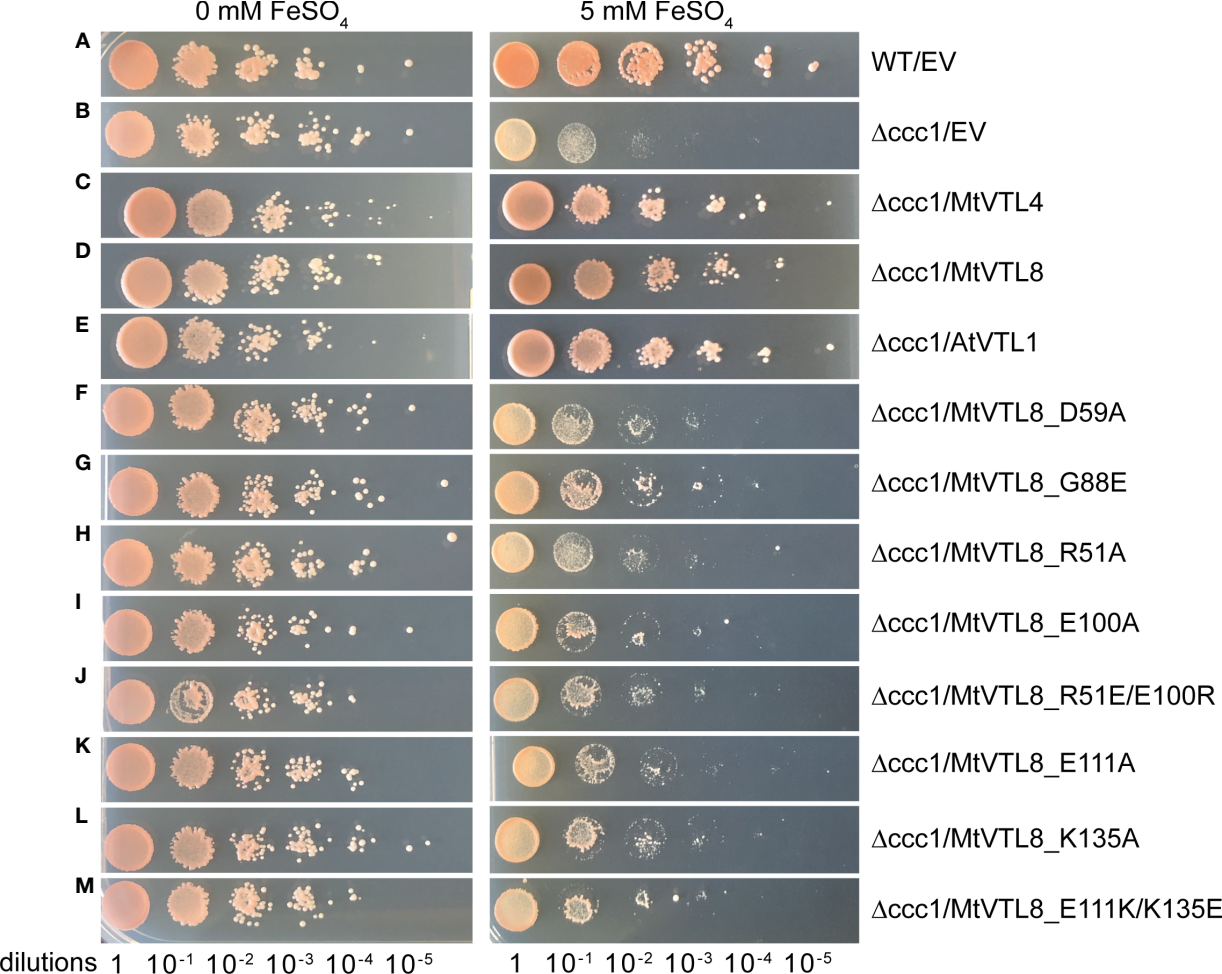
Spot assay of different mutants of *MtVTL8* expressed in the *S. cerevisiae Δccc1* strain. Spot assay with tenfold dilutions of overnight yeast cultures plated on at 0 or 5 mM ferrous sulfate (left and right panels, respectively) and incubated at 28°C for 3 days. From top to bottom: **(A)** wild-type strain DY150 transformed with empty vector (EV), **(B–M)** *Δccc1* strain transformed with EV and vector containing *MtVTL4, MtVTL8*, *AtVTL1* or specific *MtVTL8* mutant genes under the p*GAL1* promoter. *Δccc1* expressing *AtVTL1, MtVTL4* or *MtVTL8* are positive controls. Vector is pYES2/CT.

The *S. cerevisiae* wild-type DY150 and mutant *Δccc1* strains transformed with empty vector (EV) pYES2/CT individually were able to grow on selective medium without Fe^2+^ (**Figures 2A, B**, left side). Mutant *ScΔccc1* were transformed with pYES2/CT harboring the genes for *AtVTL1*, a positive control, *MtVTL4*, or *MtVTL8* were able to grow on selective medium without Fe^2+^ (**Figures 2C–E**, left side). *ScΔccc1* transformed with EV was unable to grow on the selection plates supplied with 5 mM Fe^2+^ (**Figure 2B**, right side). In contrast, the growth of the *ScΔccc1* strain in the presence of 5 mM Fe^2+^ was partially restored by heterologous expression of *AtVTL1*, a positive control, *MtVTL4*, and *MtVTL8* (**Figures 2C–E**, right side). This confirms that both MtVTL8 and MtVTL4, as well as AtVTL1, are able to transport ferrous ions out of the cytosol either to the vacuole or across the plasma membrane in yeast.

**Figure 3:**
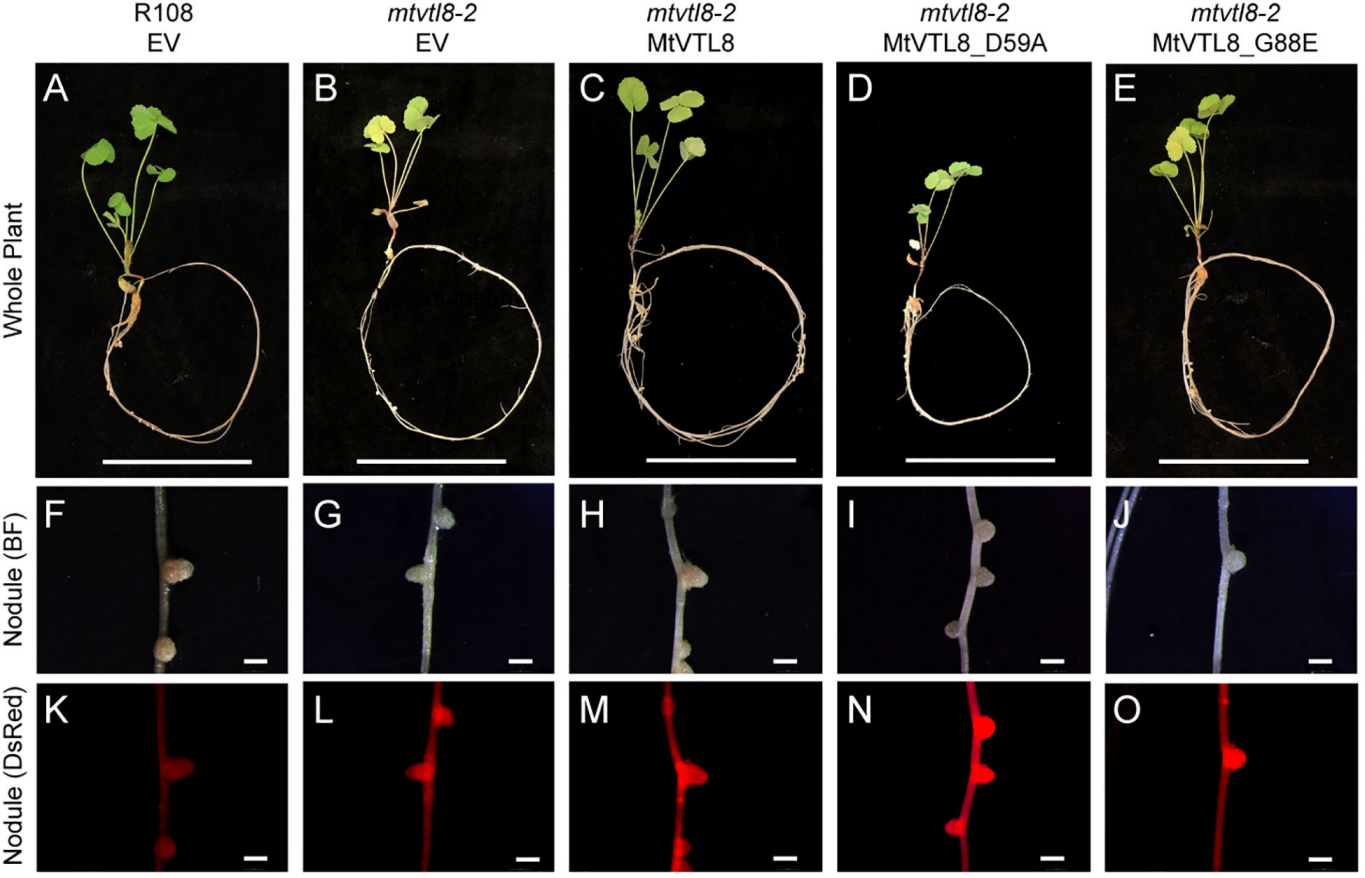
*In planta* complementation assays of the *mtvtl8-2* mutant with the wild-type *MtVTL8* and its different mutated versions affected in the transmembrane domain. **(A–E)** Images of *M. truncatula* plants transformed with different vectors expressing different mutated versions of *MtVTL8*. From left to right, **(A)** R108 plant roots transformed with empty vector (EV). **(B–E)** *Mtvtl8-2* plant roots transformed with **(B)** EV, **(C)** *MtVTL8* (WT), **(D)** *MtVTL8*_D59A, and **(E)** *MtVTL8*_G88E expressed under pMt*VTL8* promoter. **(F–J)** Bright field (BF) images of nodules corresponding to **(A–E)**. **(K–O)** DsRed fluorescence encoded by the *DsRed* gene under the p*AtUBI* constitutive promoter in the vector is observed in the transformed nodules corresponding to **(F–J).** Scale bars represent 5 cm for **(A–E)** and 1 mm for **(F–O)**.

In addition to the yeast assay, we performed *in planta* complementation using the SNF defective line *mtvtl8-2*. *In planta* complementation is a good platform to investigate plant transporters and has been used in our lab to complement *M. truncatula* plants with defective nodulation due to a mutated nitrate transporter (Yu et al., 2021). Expression of MtVTL8 driven by its 2.8 kb MtVTL8 native promoter in the mtvtl8-2 root system was accomplished by A. rhizogenes mediated hairy-root transformation (**Figures 3**). Wildtype *MtVTL8* constructs successfully rescued the defective nodule phenotype from small Fix^-^ white nodules with empty vector (**Figures 3B, G**) to wild-type like (WTL) pink nodules (**Figures 3C, H**; compare to WT with empty vector, **Figures 3A, F**). Complemented or control plants’ nodule surface areas were assessed with the aim of determining if *MtVTL8*- complemented *mtvtl8-2* nodules produced statistically similar nodule surface areas to WT R108 nodules, larger than the non-complemented *mtvtl8-2* nodules. However, none were found to have statistically different sizes (**Supplementary Figure S4**). Chlorophyll content of composite *MtVTL8*- expressing *mtvtl8-2* plants was used to assess effective nitrogen fixation. Results showed similar chlorophyll content of plants with *MtVTL8*- expressing *mtvtl8-2* roots was similar to WT R108 plants, which was markedly higher than *mtvtl8-2* plants whose roots with an empty vector-transformed (**Supplementary Figure S5**). This demonstrates that complementation (pink color in nodule and leaf chlorophyll content) can be used to assess MtVTL8 functionality *in planta*.

### *MtVLT4* and misexpression of *MtVTL8* fail to rescue *Mtvtl8*’s defective phenotype

Two *M. truncatula* CCC1/VIT1 genes, MtVTL4 and MtVTL8, are exclusively and highly expressed in nodules. Sequence alignment shows that the two proteins are 55% identical and 72% positives. Expression of MtVTL4 or MtVTL8 was found to successfully rescue the toxicity of iron in yeast *ScΔccc1* mutants (Walton et al., 2020) and confirmed in our lab (**Figures 2C, D**). These results indicate that both transporters can transport ferrous ions out of the cytosol in yeast. While *mtvtl8* mutants, either *mtvtl8-1* with a large deletion spanning *MtVTL8* and *MtVTL4* (Walton et al., 2020), or with a homozygous *Tnt1* insertion in the *MtVTL8* exon (*mtvtl8-2*) (Cai et al., 2022) display defective white nodules, Walton et al. (2020) found that plants with a *Tnt1* homozygous insertion in the *MtVTL4* exon display wild-type like nodules, with an apparent developmental delay (Walton et al., 2020). Expression of *MtVTL8* under the control of its own promoter successfully rescued the *mtvtl8-1* deletion mutant, but *MtVTL4* expression under the control of its own promoter did not have the same result (Walton et al., 2020).

Since both MtVTL4 and MtVTL8 transport Fe^2+^ in yeast, we wondered whether the failure of *MtVTL4* expression to complement *mtvtl8-1* (Walton et al., 2020) could have been caused by it being expressed at the wrong time and place in maturing nodules. To address this question, we expressed the *MtVTL4* gene driven by the constitutive *Arabidopsis thaliana* translation elongation factor (*AtEF1ɑ*) promoter (Axelos et al., 1989; Auriac and Timmers, 2007) in the roots of *mtvtl8-2*. As a control, we expressed *MtVTL8* in the same vector. The results showed that expression of neither *MtVTL4* nor *MtVTL8* driven by the *AtEF1ɑ* promoter in the roots of *mtvtl8-2* rescued the defective nodulation (**Supplementary Figures S6D, E, I, J**). While these results are not definitive for *MtVTL4*, they suggest that expression of *MtVTL8* in an inappropriate nodule location may be deleterious to nodule development. Alternatively, the *AtEF1ɑ* promoter may not express well in the area(s) of the nodule where *MtVTL8* is needed.

Because *MtVTL4* and *MtVTL8* have different expression patterns within nodules (Roux et al., 2014; Walton et al., 2020) with *MtVTL4* expressed most highly in ZII and *MtVTL8* expressed most highly in the IZ and ZIII, we wondered if expressing *MtVTL4* under *MtVTL8*’s promoter might enable *MtVTL4* to functionally complement *mtvtl8*. However, expression of *MtVTL4* under the *MtVTL8* promoter displayed defective nodulation in *mtvtl8-2* (**Supplementary Figures S7D, H**). Plants with roots transformed with *MtVTL4* had leaves containing significantly less chlorophyll compared with the positive controls (**Supplementary Figure S5**). Thus, our data suggest that MtVTL4’s localization to the plasma membrane and infection thread or other functional differences from MtVTL8 give these two transporters unique capabilities in nodules.

### Structural model of MtVTL8 and identification of essential amino acids

**Figure 4:**
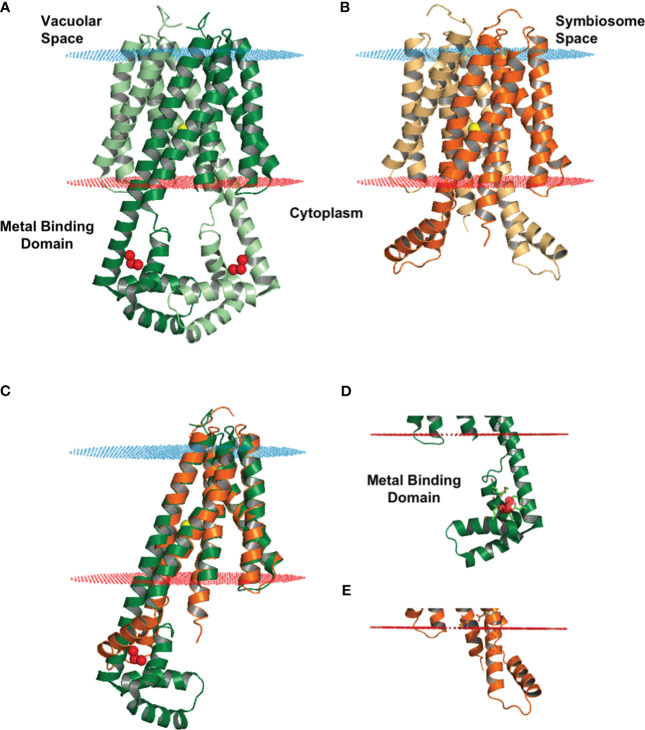
Structural model of MtVTL8 compared to the crystal structure of EgVIT1. **(A)** Crystal structure of EgVIT1, with monomer A in dark green and monomer B in light green, showing the TMD embedded in the membrane (blue and red dots) and the MBD in the cytoplasm. **(B)** AlphaFold model of MtVTL8, with monomer A in dark orange and monomer B in light orange, showing a conserved TMD and a reduced cytoplasmic region lacking a MBD. **(C)** Monomer A of MtVTL8 (dark orange) overlapped on monomer A of EgVIT1 (dark green). **(D)** Zoom into the MBD of EgVIT1 showing the side chains for the residues that bind the Fe^2+^ ions. **(E)** Zoom into the cytoplasmic region of MtVTL8 showing a shorter TMH2 and one α-helix. **(A–E)** Iron ion in the TMD is a yellow sphere; iron ions in the MBD are red spheres.

We obtained a structural model for the monomer of MtVTL8 from the AlphaFold protein structure database (Jumper et al., 2021; Varadi et al., 2022). We then produced the dimeric form (**Figure 4A**) by overlapping the monomer on chains A and B of EgVIT1, pdb 6IU3 (Kato et al., 2019). The dimeric model for MtVTL8 shows the predicted five transmembrane helices (TMHs) from TMH1 to TMH5 (**Figure 4B**) contributing to form the transmembrane domain (TMD). A cavity forms at the interface between the two monomers and in the model is open toward the cytoplasm, like in the EgVIT1 structure. The transmembrane region does not show remarkable differences between the two transporters (**Figure 4C**) (RMSD value of 1.23 for 650 atoms). The only exception is TMH2 that in the MtVTL8 model is slightly bent in the direction of TMH1 (**Figures 4D, E**). This may be due to constraints caused by a shortened sequence between TMH2 and 3. In contrast to the TMD region, structural alignment between the AlphaFold model of MtVTL8 and the crystal structure of EgVIT1 shows intriguing differences in the cytoplasmic region of the two proteins (**Figure 4C**). Like in EgVIT1, TMH2 in MtVTL8 is longer than the other TMHs and protrudes in the cytoplasm. However, in MtVTL8 the cytoplasmic portion of TMH2 is predicted to be seven residues shorter than TMH2 in EgVIT1 (**Supplementary Figure S8A**). Also, while in EgVIT1 the extended TMH2 connects with three additional cytoplasmic α-helices to form a metal binding domain (MBD) (**Figures 4A, C, D**), in MtVTL8 a shorter sequence, MtVTL8(124-138), between TMH2 and 3 is predicted to form one α-helix, H1 (**Figures 4B, C, E**). In the MtVTL8 model, TMH2 and H1 are almost parallel and are separated by a short loop of seven residues (**Figures 4B, C, E**; **Supplementary Figure S8C**). In summary, the overall topology of the transporter is changed from five transmembrane helices plus three cytoplasmic helices in EgVIT1 to five transmembrane helices plus one cytoplasmic helix in MtVTL8 (**Supplementary Figures S8B, C**).

In the EgVIT1 structure, five glutamic acid residues, Glu102 (TMH2), Glu105 (TMH2), Glu113 (H1), Glu116 (H1), and Glu153 (H3) and two methionines, Met149 and Met150 (H3), within the MBD are involved in binding and stabilizing transition metal ions like Fe^2+^ or Mg^2+^ (**Supplementary Figure S8A**, red arrowheads). Mutating any of the glutamic acids or the methionines failed to complement the growth inhibition phenotype of *Sc△ccc1* and decreased transport activity in liposomes made with the transporter mutant versions (Kato et al., 2019). Additionally, two other residues, Glu32 and Asp36 (**Supplementary Figure S8A**, red arrowheads), located at the entrance of the ion-translocation pathway on TMH1, may play a role in guiding iron ions from the MBD to the TMD. With the exception of Glu102 on TMH2 (Asp118 in MtVTL8), no amino acid residues with similar biochemical properties in similar locations are found in the cytoplasmic domain of MtVTL8 (**Supplementary Figure S8A**). This suggests that MtVTL8 is not able to bind metal ions in the cytoplasm.

We used the MtVTL8 structural model to identify residues potentially important for transport activity in VTL. After identifying residues that may have a role in metal ion binding or transport, we studied the effect of point mutations on the transport properties of MtVTL8 in complementation studies in the *mtvtl8-2* mutated plant line and in *ScΔccc1*, the iron sensitive mutant yeast strain.

### Complementation studies to test amino acids involved in the translocation pathway

In EgVIT1, two residues from each monomer in the translocation channel, Asp43 and Met80 are predicted to bind metal ions, followed by relaying the metal ions to Glu72 within the central translocation pathway (Kato et al., 2019). The three residues are all essential for EgVIT1 transport activity as demonstrated by yeast spot assay and liposomal essays. Based on sequence alignment between EgVIT1 and MtVTL8, residues Asp43 and Met80 of EgVIT1 correspond to residues Asp59 and Met96 of MtVTL8, respectively (**Supplementary Figure S8A**). However, Glu72 is not conserved in MtVTL8 as the equivalent position is occupied by a glycine, Gly88 (**Supplementary Figure S8A**).

To test its impact on the function of MtVTL8, we mutated the polar residue Asp59 to Ala to obtain the MtVTL8_D59A mutant. *A. rhizogenes-mediated* transformation of mtvtl8-2 mutant roots was used to express constructs of empty vector (EV), MtVTL8, and MtVTL8_D59A (**Figures 3A–D, F–I, K–N**). Our complementation experiments showed that *mtvtl8-2* roots transformed with the *MtVTL8_D59A* mutant gene displayed defective nodulation with white nodules (**Figures 3D, I**) compared with the pink nodules from the positive controls, wild type plant R108 transformed with EV (**Figures 3A, F**) or *mtvtl8-2* transformed with *MtVTL8* (**Figures 3C, H**). *Mtvtl8-2* plants transformed with *MtVTL8_D59A* had leaves that contained less chlorophyll compared with positive controls, and no significant difference was observed compared with the negative control (**Supplementary Figure S5**), suggesting defects in N supply. Taken together, our results indicate that *mtvtl8-2* roots transformed with *MtVTL8_D59A* showed defective SNF and did not complement the *mtvtl8-2* phenotype. Similarly, expression of the *MtVTL8_D59A* mutant failed to complement the yeast *ScΔccc1* strain (**Figure 2F**). Together these results suggest that Asp59 is essential to Fe^2+^ transport in MtVTL8.

In EgVIT1, Glu72 on TMH2 is an essential residue proposed to translocate the metal ions by displacing its bonded proton along the central ion translocation pathway (Kato et al., 2019). The corresponding residue in MtVTL8 is a glycine, Gly88. We speculated that adding back the glutamic acid in the place of Gly88 in MtVTL8 could have a positive effect on the function. Therefore, we replaced Gly88 with Glu to make the single mutant MtVT8_G88E. Expression of *MtVTL8_G88E* in *mtvtl8-2* hairy roots produced transgenic roots that displayed defective white nodules compared with the pink nodules from the positive controls (**Figures 3E, J, O**). *Mtvtl8-2* plants with roots transformed with *MtVTL8_G88E* had chlorotic leaves (**Supplementary Figure S5**). When evaluated in the yeast *ScΔccc1* mutant, the *MtVTL8_G88E* mutant failed to restore iron tolerance (**Figure 2G**). These data indicate that Gly88 is an essential residue for MtVTL8 function and mutating it to glutamic acid does not restore its putative role as proton transporter proposed for EgVIT1. Interestingly, MtVTL8 does not harbor other negatively chargeable amino acids in the substrate cavity that could potentially fulfill the same role as Glu72. These observations suggest that MtVTL8 may use a different mechanism for iron transport than that hypothesized for EgVIT1. It is also possible that another residue far from MtVTL8’s Gly88 could function in proton transport if MtVTL8 is indeed an iron-proton antiporter.

### Putative TMH1-TMH2 salt bridge formation in MtVTL8 TMD

**Figure 5:**
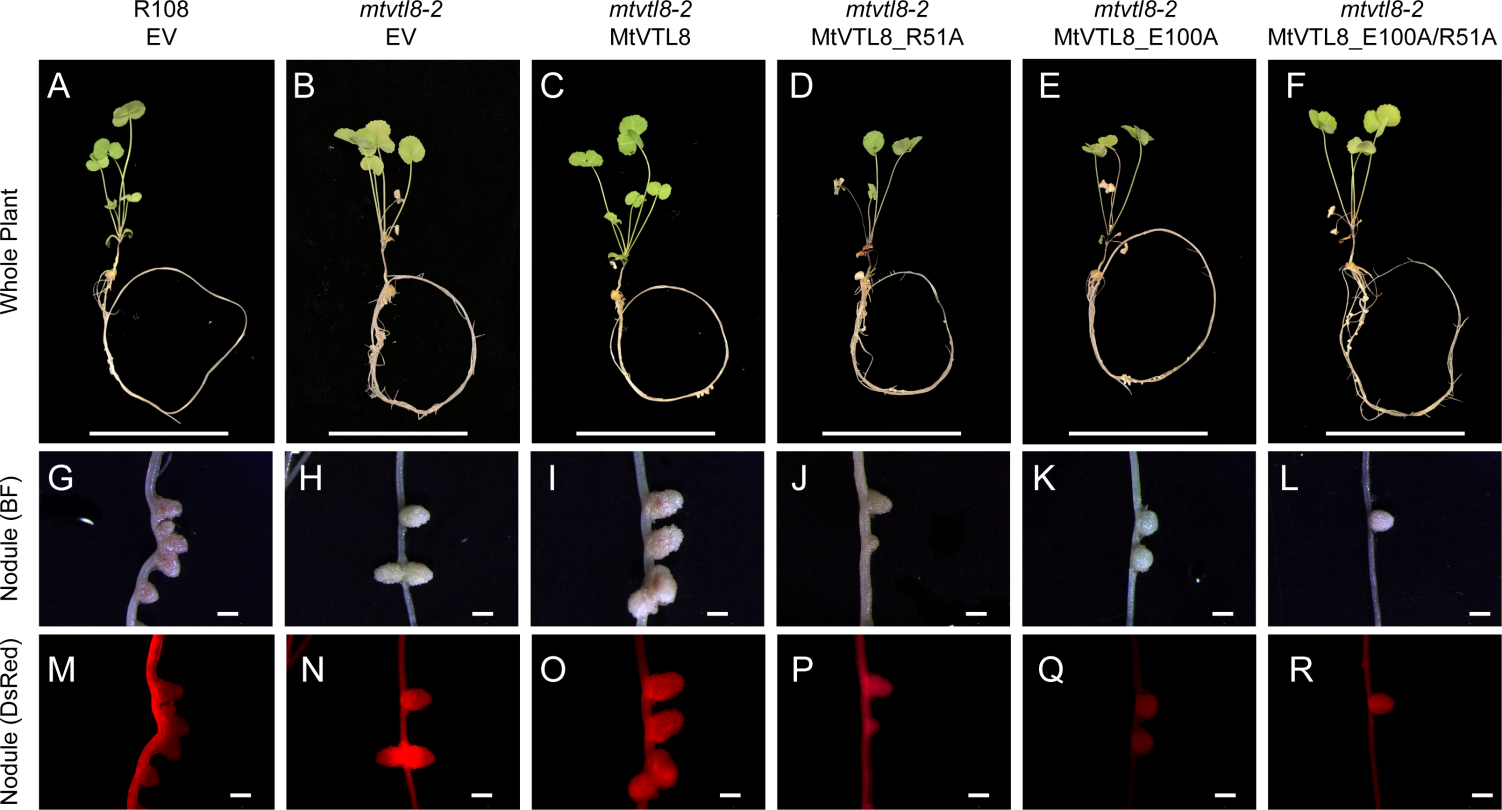
*In planta* complementation assays of the *mtvtl8-2* mutant with wild-type mutations and its different mutated versions affected in the putative TMH1-2 salt bridge. **(A–F)** Images of *M. truncatula* plants expressing different mutated versions of *MtVTL8*. From left to right, **(A)** R108 plant roots transformed with empty vector (EV); *Mtvtl8* plant roots transformed with **(B)** EV, **(C)** *MtVTL8* under p*MtVTL8* (WT), **(D)** *MtVTL8*_R51A, **(E)** *MtVTL8*_E100A, and **(F)** *MtVTL8*_R51E/E100R. **(G–L)** Bright field (BF) and **(M–R)** DsRed fluorescence images of transformed nodules corresponding to **(A–F)**. Scale bars represent 5 cm for **(A–F)** and 1 mm for **(G–R)**.

*In silico* analysis of the MtVTL8 model indicates the formation of a salt bridge in the TMD between two oppositely charged residues, Arg51 located on TMH1 and Glu100 on TMH2 (**Supplementary Figure S9A**). Alignment of MtVTL8 with EgVIT1 shows the TMH1-TMH2 salt bridge is only possible in MtVTL8 and not EgVIT1 as the corresponding amino acids in EgVIT1 are Arg35 and Gly84 (**Supplementary Figure S8A**). Based on our observation that in MtVTL8, Arg51 and Glu100 could form a salt bridge, we constructed mutants with a single mutation of Arg51 or Glu100 into Ala. Expression of *MtVTL8_R51A* or *MtVTL8_E100A* in *mtvtl8-2* hairy roots produced plants that displayed defective nodulation and symbiotic nitrogen fixation phenotypes (**Figures 5**; **Supplementary Figures S4**, **S5**). Subsequently we tried to restore the putative salt bridge by swapping the charges and we obtained the R51E/E100R double mutant. However, the *MtVTL8_R51E/E100R* mutant gene failed to rescue the *mtvtl8-2* phenotype (**Figures 5F, L**). *Mtvtl8-2* plants transformed with *MtVTL8_R51A* or *MtVTL8_E100A* or the double mutant with swapped charges had nodules with no significant difference in size compared with the positive or negative controls (**Supplementary Figure S4**), whereas the leaves contained significantly less chlorophyll compared with the positive controls (**Supplementary Figure S5**), indicating a deficiency in SNF. In yeast, neither MtVTL8 proteins with R51A, E100A, nor R51E/E100R were able to rescue the *ScΔccc1* iron transport deficiency (**Figures 2H–J**). These data suggest that these two residues are essential for MtVTL8 function and their location in the protein is important as well, likely contributing to ionic interactions other than a salt bridge between the two residues.

### MtVTL8’s putative cytoplasmic salt bridge

**Figure 6:**
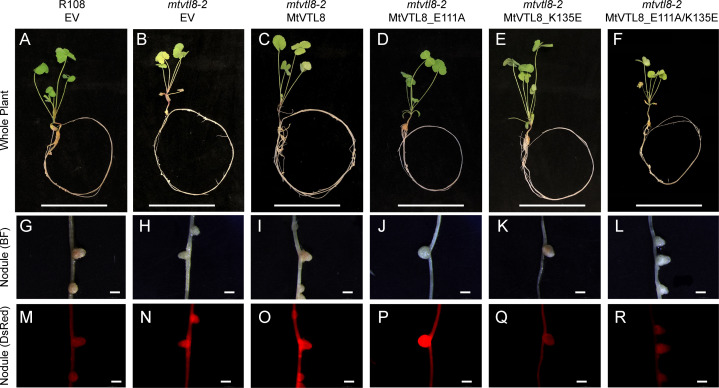
*In planta* complementation assays of the *mtvtl8-2* mutant with wild-type *MtVTL8* and its different mutated versions with mutations affected in residues capable of forming a salt bridge on the cytosolic face of the membrane. **(A–F)** Images of *M. truncatula* plants expressing different mutated versions of *MtVTL8*. From left to right, **(A)** R108 plant roots transformed with empty vector (EV); *Mtvtl8-2* plant roots transformed with **(B)** EV, **(C)** *MtVTL8* under p*MtVTL8* (WT), **(D)** *MtVTL8*_E111A, **(E)** *MtVTL8*_K135A and **(F)** *MtVTL8*_E111K/K135E under p*VTL8*. **(G–L)** Bright field (BF) and **(M–R)** DsRed fluorescence images of transformed nodules corresponding to **(A–F)**. Scale bars represent 5 cm for **(A–F)** and 1 mm for **(G–R)**.

Our model shows that the cytoplasmic portion of MtVTL8 is dramatically different from that of EgVIT1 with MtVTL8 harboring a lone α-helix MtVTL8(124-138) (H1) instead of the three α-helices as in EgVIT1 (**Figure 3**; **Supplementary Figures S8B, C**). In the model, H1 lies almost parallel to the cytoplasmic portion of TMH2. We observed that Lys135 from H1 is at a distance of 2.7 Å from Glu111 (TMH2) which is compatible with the formation of a salt bridge between the two residues (**Supplementary Figure S9**). In order to assess the role of Glu111 and Lys135, we created the single mutants *MtVTL8_E111A* and *MtVTL8_K135A* (**Figure 6**). The results showed that expression of *MtVTL8_E111A* in hairy roots of *mtvtl8-2* plants failed to rescue the defective nodulation phenotype (**Figures 6D, J**) whereas expression of *MtVTL8_K135A* successfully rescued the defective phenotype of *mtvtl8-2* (**Figures 6E, K**). Quantitation of nodule surface areas and leaf chlorophyll of composite plants showed no significant difference with the positive controls (**Supplementary Figures S4**, **S5**). To assess the restoration of the putative salt bridge between Glu111 and Lys135, we swapped the charges between the two residues creating the double mutant *K111E/E135K*. Expression of the double mutant with swapped charges failed to rescue the defective nodulation and nitrogen fixation phenotypes of *mtvtl8-2* (**Figures 6F, L**; **Supplementary Figures S4**, **S5**). This data suggests that Glu111 is an essential residue for MtVTL8 function as an Fe^2+^ transporter in yeast, whereas Lys135 is not *in planta*. It also suggests that trying to restore the putative salt bridge by swapping the charges in MtVTL8 is insufficient for function *in planta*. Similarly, when the single or double mutants were expressed in the *ScΔccc1* strain they were not able to rescue iron transport deficiency (**Figures 2K–M**).

### Phylogenetic analysis of plant VITs and VTLs and conservation of essential amino acids

A thorough phylogenetic analysis of plant CCC1/VIT members is not available. Sorribes-Dauden et al. (2020) obtained a phylogenetic tree using 771 sequences from Archaea, bacteria, fungi, and plants showing that CCC1/VIT homologs can be classified in eight different groups. Groups I and II contain only Bacteria or Archaea proteins, respectively. Fungal VITs are clustered in Groups VI, VII and VIII. Plant VITs (69 sequences) belong to Group V, plant VTLs (111 sequences) belong to Group III. Group V also includes several bacterial VITs. Based on their phylogenetic analysis, Sorribes-Dauden et al. (2020) proposed that VITs and VTLs have a different origin: VITs originated from ancestral horizontal transfers from bacteria while VTLs emerged from an archaeal lineage, with both transfers dating at the origin of the last common eukaryote ancestor.

In this paper, we expanded the phylogenetic analysis to include VIT and VTL sequences from 37 plant genomes for a total of 306 sequences (**Supplementary Table S4**). We also included two sequences from the alga *Chlamydomonas reinhardtii.* After multiple sequence alignment, we obtained a maximum-likelihood phylogenetic tree (**Supplementary Figure S10**). The phylogenetic tree supports two main clades: one clade includes transporters that harbor a MBD (purple stars in **Supplementary Figure S10**) corresponding to VITs (green labels); the other clade contains sequences that lack the MBD corresponding to VITs (orange labels). However, among VTLs, there is an exception with three VTLs that contain a MBD, MpoVTL1, PpaVTL1 and PpaVTL2 (**Supplementary Figure S10**). In addition, we divided the VITs in two subclades, Group I and II, as they display different structural features as described below.

To learn more about the conservation level of specific amino acids in VTLs and VITs and to validate the results of our mutagenesis experiments, we used our multiple sequence alignment to create separated LOGOs of specific regions for the two phylogenetic groups. We observed that residues corresponding to Asp43 and Met80 in EgVIT1 (**Supplementary Figures S11A, C**), Asp59 and Met96 in MtVTL8 (**Supplementary Figures S11B, D**) are strictly conserved in VITs and VTLS, respectively. Intriguingly, we observed that Asp72 which has been proposed to have a role in iron translocation as well as proton movement toward Asp43 (Kato et al., 2019), is only conserved in Group I VITs, while it is a Gln in the 22 VITs belonging to Group II (**Supplementary Figure S11C**). In VTLs, Asp72 is diverged to a glycine (Gly88 in MtVTL8) or other non-chargeable residue in VTLs (**Supplementary Figure S11D**).

Residues involved in metal binding in the MBD, Glu102, Glu105, Glu113, Glu116, Met149, Met150, and Glu153 are strictly conserved in Group I VITs (**Supplementary Figures S11E–G**). However, in Group II VITs, these residues are far less conserved. With the exception of Glu102 that corresponds to Asp118 in MtVTL8, the metal binding residues are absent in VTLs as they lack the MBD region. Note that TMH2 is shorter in MtVTL8 and Asp118 is the last residue of TMH2. On a side note, we should mention that the glutamic acids within the cytoplasmic MBD are conserved in PfVIT but functional expression of mutants with substitutions of glutamic acid residues in *ScΔccc1* showed iron tolerance (Sharma et al., 2021). Note that PfVIT was not included in our phylogenetic analysis. Additionally, two residues with a role in transferring iron ions from the MBD to the TMD, Glu32 and Asp36, are highly conserved in VITs, but are instead conserved non-polar residues in VTLs.

We also investigated if residues predicted to form two salt bridges, Arg51-Glu110 (TMH1-TMH2) and Glu111-Lys135 (TMH2-H1), in the MtVTL8’s AlphaFold model are conserved in VTLs and VITs. We obtained different results for the two salt bridges. Arg51 is conserved in both VITs and VTLs (**Supplementary Figures S12A, B**). However, Glu100 is only conserved in VTLs (**Supplementary Figure S12D**) and in Group II VITs (**Supplementary Figure S12C**), while it is a glycine in Group I VITs (**Supplementary Figure S12C**). Therefore, a salt bridge between TMH1 and TMH2 could only form in VTLs and Group II VITs. Finally, Glu111 (TMH2) is strictly conserved in all VTLs (**Supplementary Figure S12D**). The quality of alignment for the H1 region among VTLs was not enough to determine the conservation of Lys35 (H1).

These phylogenetic analyses reinforce the conclusions from our complementation studies with mutagenized MtVTL8 proteins. Together, they indicate that MtVTL8 and other VTLs operate by a mechanism that is distinct from that proposed for the VITs. Our analysis also indicates that a subset of VITs may have a different transport mechanism as they lack some of the structural features found in other well-characterized VITs.

## Discussion and conclusions

Iron is an essential micronutrient for human nutrition and iron-deficiency anemia affects millions of people. Biofortification of crops such as wheat, corn and legumes has been indicated as a sustainable solution to combat malnutrition including iron deficiency (Garg et al., 2018; Ofori et al., 2022). Iron transporters from the CCC1/VIT family have been used to increase iron in the endosperm of wheat and barley by overexpression of TaVIT2 (Connorton et al., 2017) and in cassava roots by overexpression of AtVIT1 (Narayanan et al., 2015; Narayanan et al., 2019). Understanding the mechanism underlying iron transport by iron transporters including VTLs can result in future beneficial application for agronomic biofortification. Equally important is understanding the mechanisms of SNF that contribute to sustainable agriculture.

Our results show the spatial patterns of *MtVTL8*-promoter directed expression in roots in developing and mature nodules, with expression detected as early as 5 dpi in nodule primordia (**Figure 1**; **Supplementary Figures S1**, **S2**). The results in mature nodules (**Figure 1**) confirm previous results obtained by laser-capture dissection RNAseq for WT *M. truncatula* nodules (Roux et al., 2014) showing expression in the proximal areas of ZII, IZ and ZIII. Expression of *MtVTL8* was not observed in the vascular system (**Figure 1**; **Supplementary Figures S1A, E**, **S2A, B** **,** **S3A, B**). These results are somewhat different from those obtained in a study of the homologous *GmVTL1a* gene, in which expression was observed in cells surrounding the nodule vasculature and in infected nodule cells (Brear et al., 2020). In mutant *mtvtl8-2* nodules, expression was similar to WT (**Supplementary Figures S2**, **S3** compare to **Figure 1** and **Supplementary Figure S1**). It was expressed early in nodulation, in 5 dpi nodule primordia (**Supplementary Figures S2H–J**) and in the distal zones of the nodule, but much lower in the proximal zones, demarking the nodule zones where MtVTL8 is essential for nodule function (**Supplementary Figures S1**–**3**). This suggests that MtVTL8 is dispensable for early nodule development and only becomes essential after the bacteroids are enclosed by symbiosome membranes.

Because both *MtVTL4* and *MtVTL8* are expressed solely in nodules with different spatial expression patterns as assessed by RNAseq (Roux et al., 2014; Walton et al., 2020), with *MtVTL4* only expressed in cells where rhizobia are being released into symbiosomes, we were curious to see if expression of *MtVTL4* using *MtVTL8 cis* elements would complement the defect in *mtvtl8-2*; i.e., does MtVTL4 have similar functionality if expressed in place of MtVTL8? The p-*MtVTL8-MtVTL4* construct did not complement *mtvtl8-2* (**Supplementary Figure S7**), while expression of either gene in the yeast *△ccc1* mutant rescued the iron toxicity phenotype (**Figures 2C, D**), leading us to propose that MtVTL4 and MtVTL8 have distinct functions beyond iron transport per se. We note that our results may be caused by intracellular localization of MtVTL4 vs. MtVTL8 as well as differences in function between the two proteins.

**Figure 7:**
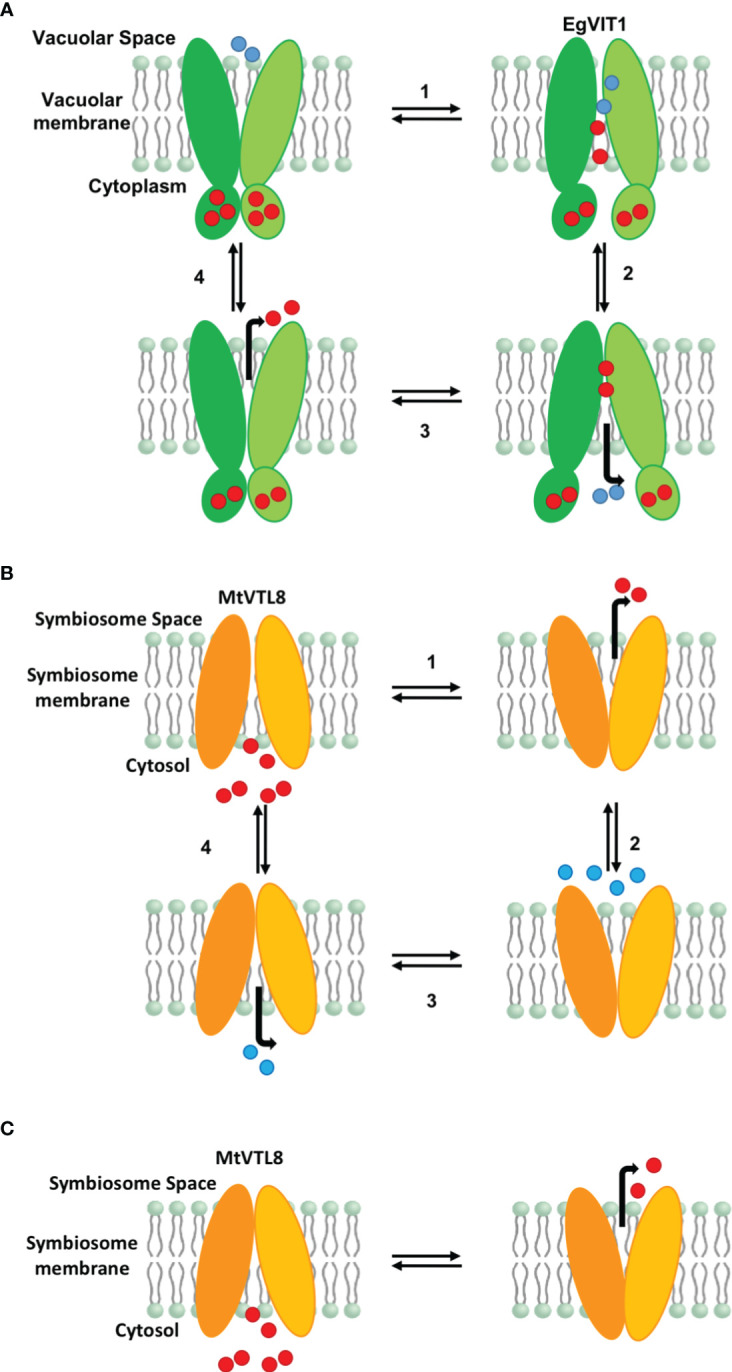
Proposed models for iron transport in plant VITs and VTLs. **(A)** Proposed model for EgVIT1 as a Fe^2+^/H^+^ antiporter that utilizes a multiple-site alternating-access mechanism. Iron ions are captured in the cytosol and stored in the MBD. Iron ions and protons travel in opposite directions through the transporter simultaneously during the alternating access cycle. Protons enter the transport channel with the transporter in the conformation open toward the vacuolar space. Iron ions enter the transport channel in the cytosol open conformation. Iron ions protons are exchanged in the cavity and protons are released in the cytosol. When the transporter opens again toward the vacuolar space, iron ions are released. **(B)** Proposed model for MtVTL8 as a Fe^2+^/H^+^ antiporter that utilizes a single-site alternating-access mechanism. Iron ions and protons do not travel simultaneously in the transport channel. As it lacks a MBD, MtVTL8 does not store iron ions. Instead iron ions enter the transporter channel when the protein is open toward the cytosol and are then released in the symbiosome space in the inward open conformation. Protons enter the transport channel and are released when the transporter opens again toward the cytoplasm. **(C)** Proposed model for MtVTL8 as a Fe^2+^ uniporter. In this model MtVTL8 operates as a uniporter and there is no transport of protons. **(A, B)** Numbers corresponds to the proposed order of events. EgVIT1 in green, MtVTL8 in orange. Iron ions are shown as red spheres, protons as blue spheres.

Functional studies for PtVIT and EgVIT1 (Labarbuta et al., 2017; Kato et al., 2019) showed they are Fe^2+^/H^+^ antiporters that exchange metal ions and protons on the opposite sides of lipid membranes. The solved crystal structure for EgVIT1, which captured the inside-open conformation, allowed the identification of essential amino acids for the transport of iron/protons (Kato et al., 2019). A model has been proposed in which the proton/iron transport cycle starts on the cytosolic side of the membrane where metal ions are captured by the MBD (**Figure 7A**). Conserved residues in the MBD and the TMD substrate channel entrance provide a pathway for the iron ions to translocate from the MBD to the TMD. Metal ions are transferred to a metal binding pocket in the TMD that is alternatively exposed to either side of the membrane. Two chargeable amino acids in the TMD, Asp43 and Glu72 are the initial iron and proton binding sides, respectively. After an exchange of position happens, irons are released in the vacuolar space and protons in the cytosol. We note that although most antiporters utilize a so-called single-site alternating-access mechanism, where the transporter can only associate with one substrate at a time, there are some examples of antiporters where ion and substrate binding sites were not overlapping, e.g. in the NorM transporters (Lu et al., 2013; Claxton et al., 2021). Despite the proposed model, many details of the mechanism of transport for EgVIT1 and for VITs in general are not fully understood. For instance, although a cooperative action is expected to take place between the TMD and the auxiliary cytoplasmic MBD, the role of the latter in transport is still not clear. Also, the stoichiometry of transport is not known.

In the absence of a crystal structure for VTLs, we obtained a model for MtVTL8. Using such model as a guide we identified amino acids potentially involved in iron transport in MtVTL8. We demonstrated that when mutated, the resulting protein cannot restore SNF in plants or iron tolerance in yeast confirming our hypotheses. We proposed that MtVTL8 could rely on a different transport mechanism from that proposed for VITs and suggested two alternative mechanisms. As discussed previously, VTLs, including MtVTL8, lack the cytoplasmic MBD and the amino acids that can capture metal ions in the cytoplasm. Additionally, they lack amino acids that guide the metal ions toward the binding pocket and an essential glutamic acid residue, Glu72, that has been indicated to work as relay for proton-iron exchange in the TMD. In VTLs, Glu72 is substituted with a glycine (Gly88 in MtVTL8) but our studies have shown that the MtVTL8_Gly88Glu mutant is not an active transporter (**Figures 2H**,**3E, J, O**). Based on these observations, we have two working hypotheses. Our first hypothesis is that MtVTL8 is a Fe^2+^/H^+^ antiporter that uses the symbiosome proton gradient to fuel Fe^2+^ transport (**Figure 7B**). In this scenario, in MtVTL8, Asp59, the only membrane-embedded charged residue, is a common binding site for substrate and protons, and the occupancy of this site is mutually exclusive (**Figure 7B**). This is agreement with what was proposed for other antiporters and called single-site alternating-access mechanism of transport (Yerushalmi and Schuldiner, 2000; Schuldiner, 2014). Another possibility is that MtVTL8 uses an antiporter mechanism similar to that proposed to EgVIT1 but uses an amino acid residue distant from Gly88 as a proton acceptor. Because we lack experimental evidence that MtVTL8 is an Fe^2+^/H^+^ antiporter, there exists the less-likely possibility that MtVTL8 and other VTLs may operate as Fe^2+^ efflux channels instead of being active as active Fe^2+^/H^+^ antiporters (**Figure 7C**). In this model, Fe^2+^ binds to the conserved Asp59 inside the channel and is transported without an exchange of protons. This hypothetical mechanism spares the pH gradient across the symbiosome membrane required for nitrogen fixation (Udvardi and Day, 1989; Pierre et al., 2013). The gradient is essential for the import of dicarboxylates for SNF and believed to be important for the import of other molecules. In this less-likely scenario, the recently identified sequestration of heme by *M. truncatula’*s NCR247 that overrides the bacteroids’ negative Fe^2+^ regulation of Fe^2+^ import (Sankari et al., 2022) might result in a lowered Fe^2+^ concentration in the symbiosome space. That lowered Fe^2+^ concentration might be low enough to provide a sufficient driving force for Fe^2+^ movement across the symbiosome membrane from the cytosol without requiring an additional source of energy for transport. Additional studies are needed to address these hypotheses including molecular dynamics calculations, transport assays and experimental structures for VTLs.

## Data availability statement

The original contributions presented in the study are included in the article/**Supplementary Material**. Further inquiries can be directed to the corresponding author.

## Author contributions

JC: Conceptualization, Data curation, Formal analysis, Investigation, Methodology, Visualization, Writing – original draft. AL: Conceptualization, Data curation, Formal analysis, Investigation, Methodology, Validation, Writing – original draft, Writing – review & editing. RD: Conceptualization, Data curation, Funding acquisition, Investigation, Methodology, Project administration, Resources, Supervision, Writing – original draft, Writing – review & editing.
