# Supplementary material for: Expression and mutagenesis studies in the Medicago truncatula iron transporter MtVTL8 confirm its role in symbiotic nitrogen fixation and reveal amino acids essential for transport
Source: Front Plant Sci. 2024 Jan 4;14:1306491. doi: 10.3389/fpls.2023.1306491 (PMC10794610; doi:10.3389/fpls.2023.1306491)
Supplement: Supplementary file 1 [file DataSheet_1.zip › Supplementary Tables and Figures.pdf]

**Supplementary data for Cai et al. Expression and mutagenesis studies in the *Medicago truncatula* iron transporter MtVTL8 confirm its role in symbiotic nitrogen fixation and reveal amino acids essential for transport.**

**Supplementary Table S1. Primers list.**

**Supplementary Table S2. Plasmids list.**

**Supplementary Table S3. Construction of mutant versions of *MtVTL8*.**

**Supplementary Table S4. Sequences of plant VITs and VTLs used to construct phylogeny. (Presented as a separate Excel file)**

**Supplementary Figure S1. Expression of p*MtVTL8*-GUS in *M. truncatula* R108.**

**Supplementary Figure S2. Expression of p*MtVTL8-GUS* in *M. truncatula vtl8-2*.**

**Supplementary Figure S3. p*MtVTL8-GUS* is expressed in zone II to zone IV in both infected and uninfected cells of *mtvtl8-2*.**

**Supplementary Figure S4. Nodule surface area of *in planta* site mutagenesis complementation in composite plants.**

**Supplementary Figure S5. Chlorophyll content in leaves from composite plants in the *in planta* site mutagenesis complementation assessment.**

**Supplementary Figure S6. *In planta* expression of *MtVTL4* and *MtVTL8* driven by the *AtEF1a* promoter fails to complement *mtvtl8-2* composite plants.**

**Supplementary Figure S7. *In planta* expression of *MtVTL4* driven by the *MtVTL8* promoter fails to complement *mtvtl8-2* composite plants.**

**Supplementary Figure S8. Sequence alignment and topology of MtVTL8 and EgVIT1.**

**Supplementary Figure S9. Putative salt bridges in the AlphaFold model of MtVTL8.**

**Supplementary Figure S10. Phylogenetic tree of plant CCC1/VIT family members.**

**Supplementary Figure S11. LOGOs of conservation of VIT and VTL residues involved in metal binding and transport.**

**Supplementary Figure S12. LOGOs of conservation of residues predicted to form two salt bridges in the MtVTL8's AlphaFold model**.

**Supplementary Table 1. Primers list.** Sequences of primers used for cloning, mutagenesis and sequencing. Mutated codons in bold.

| Primer Name | Sequence (5'-3') | Purpose |
|---|---|---|
| JYC05-F1 | ACCAACCACAACGGCGCGCCCTCGAGGAAGT AAAGGGTCAAGAAGAGA | Cloning MtVTL8 promoter |
| JYC05-R1 | CATATTGTACCAACGGCCATACTAGTTGGTAC TAAAAAAAAATAAATTAAGAAAAAGAA | Cloning MtVTL8 promoter |
| JYC05-F2 | TTTATTTTTTTTAGTACCAACTAGT*ATGGCCG TTGGTACAATATGT* | Cloning MtVTL8 CDS |
| JYC05-R2 | CTAAAAATGAGGGACAACATGGTACCTCA*AAT TTCCAAATCCAAACCACT* | Cloning MtVTL8 CDS |
| JYC05-F3 | GTTTGGATTTGGAAATTTGAGGTACCATGTTG TCCCTCATTTTTAGTTAATTGT | Cloning MtVTL8 3'UTR |
| JYC05-R3 | ATCTGATCCAAGCTCAAGCTAAGCTTGTACTA TTAAGTCGGTTTATTGATGTTTCC | Cloning MtVTL8 3'UTR |
| JYC13-F | GCATGGACGAGCTGTACAAGGGTACCATG GCCGTTGGTACAATATGT | Cloning pJYC13 |
| JYC13-R | TGCGGACTCTAGCATGGCCGGGTACCTCA AATTTCCAAATCCAAACCACTA | Cloning pJYC13 |
| JYC15-F | TTTATTTTTTTTAGTACCAACTAGTATGTTA CGTCCTGTAGAAACCCC | Cloning pJYC15 |
| JYC15-R | CTAAAAATGAGGGACAACATGGTACCTCAT TGTTTGCCTCCCTGCT | Cloning pJYC15 |
| JYC16-F | ATTTATTTTTTTTAGTACCAACTAGT*ATGGCC GTTGGTACAATATGTGATT* | Cloning pJYC16-23 |
| JYC16-R | ATTAACTAAAAATGAGGGACAACATGGTACC TC*AAATTTCCAAATCCAAACCAC* | Cloning pJYC16-23 |
| JYC16-R2 | ATACGGCTGC**AGC**AAGCCATTGAGCCCTTTGC C | Mut R51A (pJYC16) |
| JYC16-F2 | TCAATGGCTT**GCT**GCAGCCGTATTAGGAGCTA ATGATG | Mut R51A (pJYC16) |
| JYC17-R2 | GAAACTAATCC**AGC**ATTAGCTCCTAATACGGC TGCG | Mut D59A (pJYC17) |
| JYC17-F2 | TAGGAGCTAAT**GCT**GGATTAGTTTCTGTTGCT TCACTAATGAT | Mut D59A (pJYC17) |

| | | |
|---|---|---|
| JYC18-R2 | CCTGCAATTAA**TTC**CGCAAAACCCGCGACTAAC | Mut G88E (pJYC18) |
| JYC18-F2 | CGGGTTTTGCG**GAA**TTAATTGCAGGAGCATGTGGTATGG | Mut G88E (pJYC18) |
| JYC19-R2 | ACAGAAACAAA**TGC**TCCTATTGCCATACCACATGCTC | Mut E100A (pJYC19) |
| JYC19-F2 | TGGCAATAGGA**GCA**TTTGTTTCTGTGTACACACAATATGAAGTTG | Mut E100A (pJYC19) |
| JYC20-R2 | ATTTGACCAAT**TGC**AACTTCATATTGTGTGTACACAGAAACAAAT | Mut E111A (pJYC20) |
| JYC20-F2 | AATATGAAGTT**GCA**ATTGGTCAAATGATGAGAGATTTAGGAACA | Mut E111A (pJYC20) |
| JYC21-R2 | ATGATCTCCT**TGC**CTCCAACTCAATCTCCAATTCTTTCTCTT | Mut K135A (pJYC21) |
| JYC21-F2 | TTGAGTTGGAG**GCA**AGGAGATCATTGCCTAATCCATTGC | Mut K135A (pJYC21) |
| JYC22-R2 | ATACGGCTGC**TTC**AAGCCATTGAGCCCTTTGCC | Mut R51E (pJYC22) |
| JYC22-F2 | TCAATGGCTT**GAA**GCAGCCGTATTAGGAGCTAATGATG | Mut R51E (pJYC22) |
| JYC22-R3 | CAGAAACAAA**GCG**TCCTATTGCCATACCACATGCTC | Mut E100R (pJYC22) |
| JYC22-F3 | GGCAATAGGA**CGC**TTTGTTTCTGTGTACACACAATATGAAGTTG | Mut E100R (pJYC22) |
| JYC23-R2 | TTTGACCAAT**TTT**AACTTCATATTGTGTGTACACAGAAACAAATTC | Mut E111K (pJYC23) |
| JYC23-F2 | CAATATGAAGTT**AAA**ATTGGTCAAATGATGAGAGATTTAGGAACA | Mut E111K (pJYC23) |
| JYC23-R3 | ATGATCTCCT**TTC**CTCCAACTCAATCTCCAATTCTTTCTCTT | Mut K135R (pJYC23) |
| JYC23-F3 | ATTGAGTTGGAG**GAA**AGGAGATCATTGCCTAATCCATTGC | Mut K135R (pJYC23) |
| JYC10.FOR | GACTCACTATAGGGAATATTAAGCTTATGGCCGTTGGTACAATATGT | Cloning MtVTL8 from pJYC05 (WT) or pJYC16-23 (mutants) |

| | | |
|---|---|---|
| JYC10.REV | CACTGGCGGCCGTTACTAGTGGATCCTCAAATTTCCAAATCCAAACCACTA | Cloning MtVTL8 from pJYC05 (WT) or pJYC16-23 (mutants) |
| JYC24-F | TTTATTTTTTTTAGTACCAACTAGTATGAAGAGACTGTGTTTCTACATGC | Cloning pJYC24 |
| JYC24-R | CTAAAAATGAGGGACAACATGGTACCTCAAAGTGAACTATAGCCAACTAATTTGG | Cloning pJYC24 |
| YJYC24-F | GACTCACTATAGGGAATATTAAGCTTATGAAGAGACTGTGTTTCTACATGC | Cloning pYJYC24 from JYC24 |
| YJYC24-R | CACTGGCGGCCGTTACTAGTGGATCCTCAAAGTGAACTATAGCCAACTAATTTGG | Cloning pYJYC24 from JYC24 |
| JYC11-F | GACTCACTATAGGGAATATTAAGCTTATGGAATCACACAACGTGAGC | Cloning pYJYC11 |
| JYC11-R | CACTGGCGGCCGTTACTAGTGGATCCTCACAGACTATGTGTCCCAATCA | Cloning pYJYC11 |
| SP05-R | AGCTCCTAATACGGCTGCG | Sequencing pJYC05, JYC15, pJYC16-23 |
| SP05-F | CAGACCAATCACACATTCATG | Sequencing pJYC05, JYC15, pJYC16-23 |
| SP05-R2 | AACTAGCTATTGCCATAATCG | Sequencing pJYC05 |
| SP05-F2 | GTTCCTTTACTTAGTGGTTCA | Sequencing pJYC05 |
| SP05-R3 | TAGTTCATCCTAGCGTTTGC | Sequencing pJYC05 |
| SP05-R4 | CCGTTATTCTAATAAACGCTC | Sequencing pJYC05 |
| S03-F | GGGAGGCTGTGGAAGAAC | Sequencing pJYC05 |
| SP13-F | ACGAGAAGCGCGATCACAT | Sequencing pJYC13 |
| S02R | GAACCCTAATTCCCTTATCTG | Sequencing pJYC13 |
| S05-R | CTGCATCGGCGAACTGATC | Sequencing pJYC16-23 |
| SP15-F2 | CAACTGGACAAGGCACTAG | Sequencing pJYC16-23 |
| SP10-F | CCTCTATACTTTAACGTCAAG | Sequencing pYJYC10/11/16-23 |
| SP10-R | GCGTGAATGTAAGCGTGAC | Sequencing pYJYC10/11/16-23 |

**Supplementary Table 2. Plasmids list.**

| Name | Construct | Purpose | Backbone | Selection |
|---|---|---|---|---|
| pMU14 | Empty vector | control | pMU14 | $Sm^R$, $Sp^R$ |
| pJYC13 | MU14 w/o DsRed-35S-GFP-MtVTL8 | *N. benthamiana* protein localization assay | pMU14 | $Sm^R$, $Sp^R$ |
| pJYC15 | MU14-p*MtVTL8-GUS* | MtVTL8-GUS for expression localization assay | pMU14 | $Sm^R$, $Sp^R$ |
| pJYC05 | MU14-p*MtVTL8-MtVTL8*-3'UTR | complementation assay for *Mtvtl8 in planta* | pMU14 | $Sm^R$, $Sp^R$ |
| pJYC15 | MU14-p*MtVTL8-GUS* | MtVTL8-GUS for expression localization assay | pMU14 | $Sm^R$, $Sp^R$ |
| pJYC16 | MtVTL8_R51A | Mutagenesis assay for *Mtvtl8 in planta* | pMU14 | $Sm^R$, $Sp^R$ |
| pJYC17 | MtVTL8_D59A | Mutagenesis assay for *Mtvtl8 in planta* | pMU14 | $Sm^R$, $Sp^R$ |
| pJYC18 | MtVTL8_G88E | Mutagenesis assay for *Mtvtl8 in planta* | pMU14 | $Sm^R$, $Sp^R$ |
| pJYC19 | MtVTL8_E100A | Mutagenesis assay for *Mtvtl8 in planta* | pMU14 | $Sm^R$, $Sp^R$ |
| pJYC20 | MtVTL8_E111A | Mutagenesis assay for *Mtvtl8 in planta* | pMU14 | $Sm^R$, $Sp^R$ |
| pJYC21 | MtVTL8_K135A | Mutagenesis assay for *Mtvtl8 in planta* | pMU14 | $Sm^R$, $Sp^R$ |
| pJYC22 | MtVTL8_R51E/E100R | Mutagenesis assay for *Mtvtl8 in planta* | pMU14 | $Sm^R$, $Sp^R$ |
| pJYC23 | MtVTL8_E111K/K135E | Mutagenesis assay for *Mtvtl8 in planta* | pMU14 | $Sm^R$, $Sp^R$ |
| pJYC24 | MU14-p*MtVTL8-MtVTL4*-3'UTR | complementation assay for *Mtvtl4 in planta* | pMU14 | $Sm^R$, $Sp^R$ |
| pJYC10 | pYES2/CT-MtVTL8 | complementation assay in yeast Δ*ccc1* | pYES2/CT | $Amp^R$, Ura3 |

| | | | | |
|---|---|---|---|---|
| pJYC11 | pYES2/CT-AtVTL1 | complementation assay in yeast Δ*ccc1* | pYES2/CT | $Amp^R$, Ura3 |
| pYJYC24 | pYES2/CT-MtVTL4 | complementation assay in yeast Δ*ccc1* | pYES2/CT | $Amp^R$, Ura3 |
| pYJYC16 | MtVTL8_R51A | Mutagenesis assay in yeast Δ*ccc1* | pYES2/CT | $Amp^R$, Ura3 |
| pYJYC17 | MtVTL8_D59A | Mutagenesis assay in yeast Δ*ccc1* | pYES2/CT | $Amp^R$, Ura3 |
| pYJYC18 | MtVTL8_G88E | Mutagenesis assay in yeast Δ*ccc1* | pYES2/CT | $Amp^R$, Ura3 |
| pYJYC19 | MtVTL8_E100A | Mutagenesis assay in yeast Δ*ccc1* | pYES2/CT | $Amp^R$, Ura3 |
| pYJYC20 | MtVTL8_E111A | Mutagenesis assay in yeast Δ*ccc1* | pYES2/CT | $Amp^R$, Ura3 |
| pYJYC21 | MtVTL8_K135A | Mutagenesis assay in yeast Δ*ccc1* | pYES2/CT | $Amp^R$, Ura3 |
| pYJYC22 | MtVTL8_R51E_E100R | Mutagenesis assay in yeast Δ*ccc1* | pYES2/CT | $Amp^R$, Ura3 |
| pYJYC23 | MtVTL8_E111K_K135E | Mutagenesis assay in yeast Δ*ccc1* | pYES2/CT | $Amp^R$, Ura3 |

**Supplementary Table S3. Construction of mutant versions of *MtVTL8*.** Primers used in construction of each mutant version of *MtVTL8*.

| **Mutation** | **Fragment** | **Primer 1** | **Primer 2** |
|---|---|---|---|
| *MtVTL8_R51A* | JYC16-1 | JYC16F | JYC16R2 |
| | JYC16-2 | JYC16F2 | JYC16R |
| *MtVTL8_D59A* | JYC17-1 | JYC16F | JYC17R2 |
| | JYC17-2 | JYC17F2 | JYC16R |
| *MtVTL8_G88E* | JYC18-1 | JYC16F | JYC18R2 |
| | JYC18-2 | JYC18F2 | JYC16R |
| *MtVTL8_E100A* | JYC19-1 | JYC16F | JYC19R2 |
| | JYC19-2 | JYC19F2 | JYC16R |
| *MtVTL8_E111A* | JYC20-1 | JYC16F | JYC20R2 |
| | JYC20-2 | JYC20F2 | JYC16R |
| *MtVTL8_K135A* | JYC21-1 | JYC16F | JYC21R2 |
| | JYC21-2 | JYC21F2 | JYC16R |
| *MtVTL8_R51E/E100R* | JYC22-1 | JYC16F | JYC22R2 |
| | JYC22-2 | JYC22F2 | JYC22R3 |
| | JYC22-3 | JYC22F3 | JYC16R |
| *MtVTL8_E111K/K135E* | JYC23-1 | JYC16F | JYC23R2 |
| | JYC23-2 | JYC23F2 | JYC23R3 |
| | JYC23-3 | JYC23F3 | JYC16R |

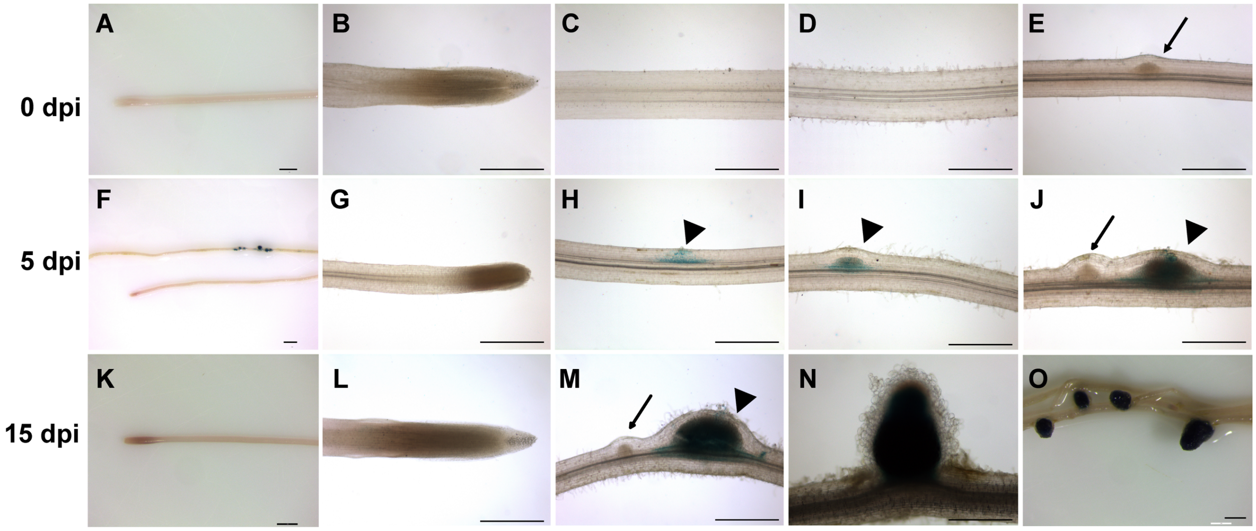


**Supplementary Figure S1. Expression of *GUS* driven by the *MtVTL8* promoter in *M. truncatula* R108.** *GUS* expression under the control of the *MtVTL8* promoter (p*MtVTL8*) was visualized using light microscopy. Roots were analyzed at different time points post inoculation with *S. meliloti* Rm41. (**A-E**) p*MtVTL8-GUS* expression at 0 dpi. (**F-J**) p*MtVTL8-GUS* expression at 5 dpi. (**K-O**) p*MtVTL8-GUS* expression at 15 dpi. Arrows indicate lateral root; arrow heads indicate nodule primordia and nodules. Scale bars represent 1 mm.

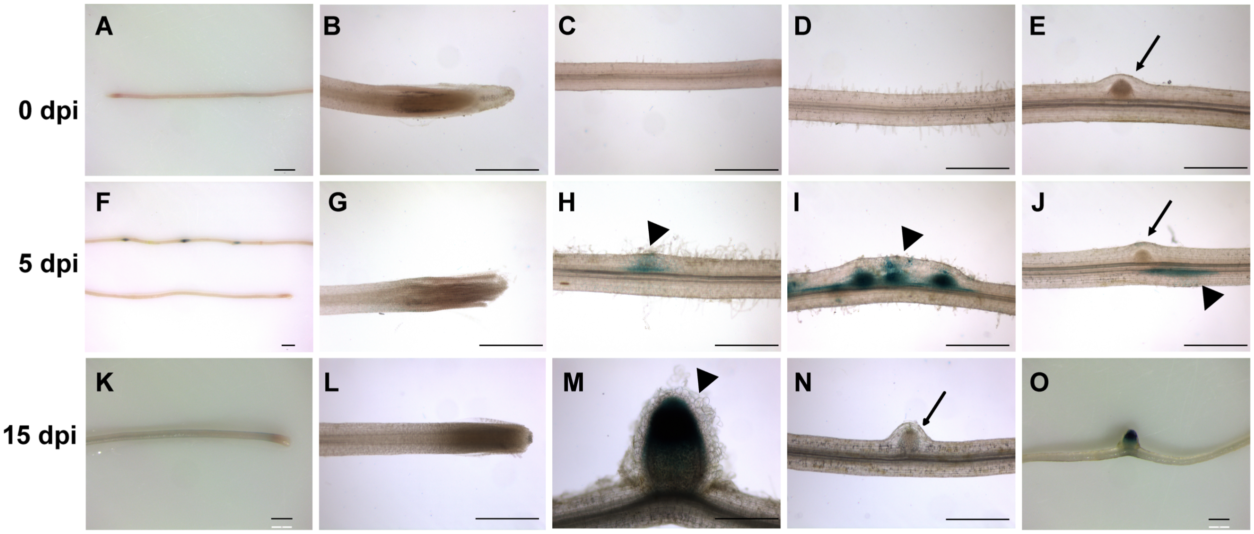


**Supplementary Figure S2. Expression of p*MtVTL8-GUS* in *M. truncatula vtl8-2*.** GUS was visualized using light microscopy. Roots were analyzed at different time points post inoculation. p*MtVTL8-GUS* expression in root systems at 0 dpi (**A-E**), 5 dpi (**F-J**), and 15 dpi (**K-O**). Arrows indicate lateral roots. Arrow heads indicate nodule primordia and nodules. Scale bars represent 1 mm.

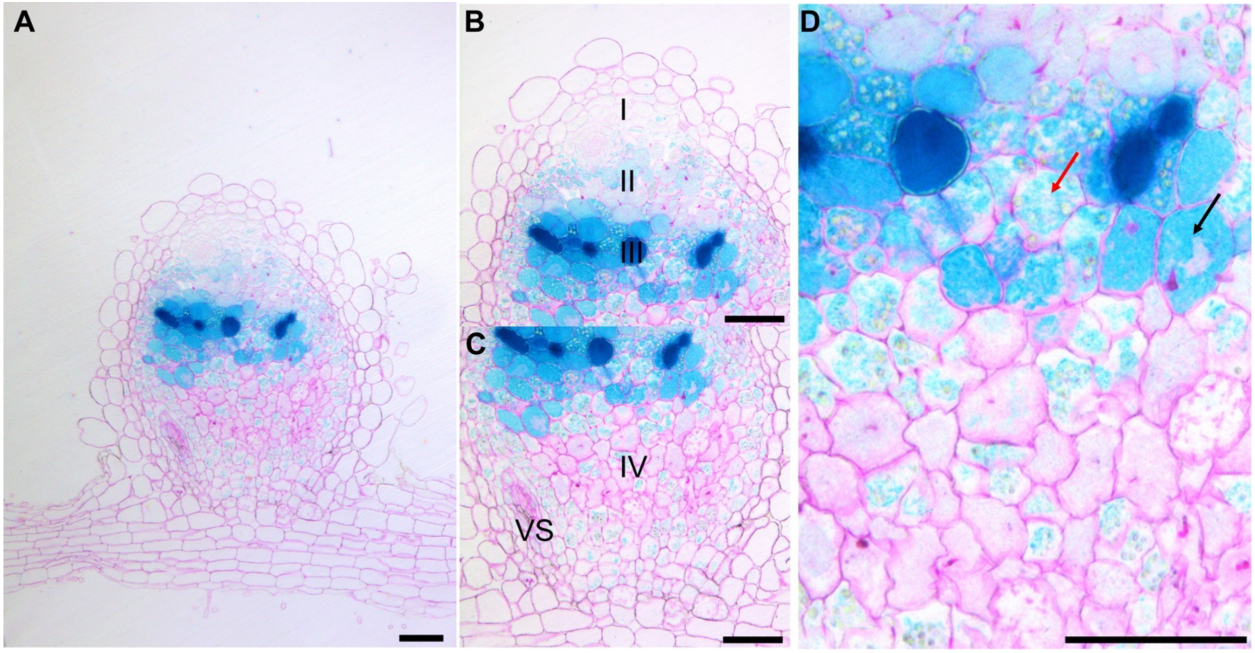


**Supplementary Figure S3. p*MtVTL8-GUS* is expressed in zone II to zone IV in both infected and uninfected cells of *mtvtl8-2*.** (**A**) Expression of p*MtVTL8-GUS* in *mtvtl8-2* nodule at 15 dpi with *S. meliloti* Rm41. (**B-D**) Expression of p*MtVTL8-GUS* from Zone I to Zone III. Blue color indicates the expression of *GUS* encoding β-glucuronidase enzyme hydrolyzing X-Gluc into diX-indigo, displaying blue color under light microscope. VS, vasculature. I, zone I, meristem zone. II, zone II, infection zone. III, zone III, fixation zone. IV, senescent zone. The black arrow indicates infected cells. The red arrow indicates uninfected cells with no rhizobia inside. Scale bars represent 0.1 mm.

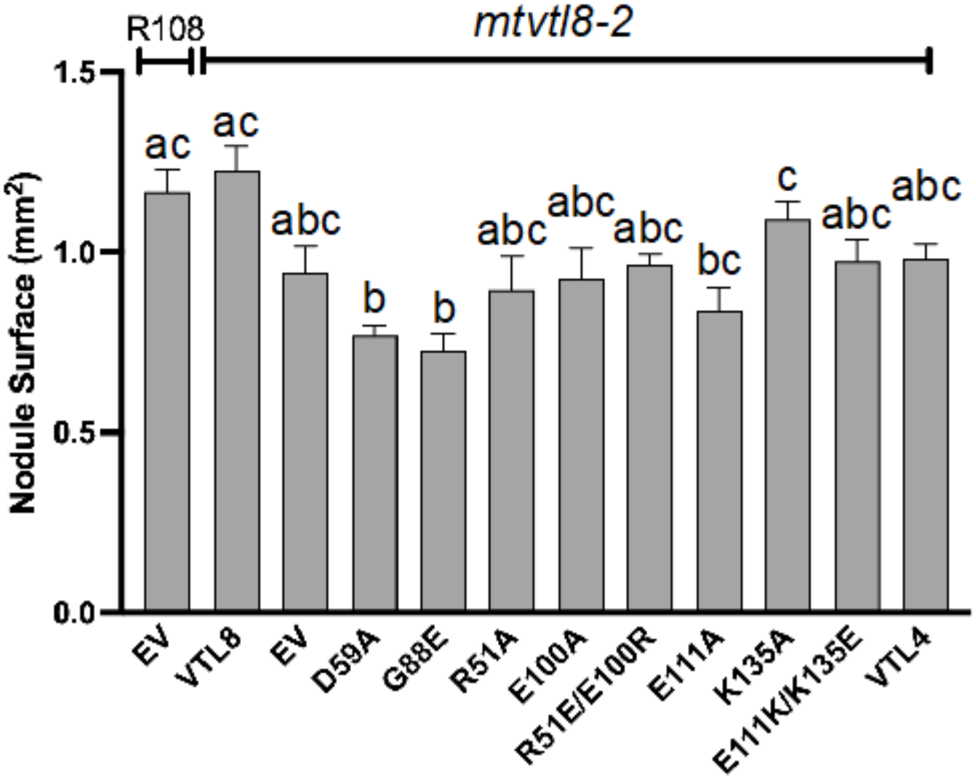


**Supplementary Figure S4. Nodule surface area of composite plants complemented with various constructs.**

The nodule surface areas of R108 and *mtvtl8-2* plants transformed with empty vector (EV), vector expressing *MtVTL8*, different indicated mutants of *MtVTL8*, or *MtVTL4*. Nodule surface areas were recorded at 15 dpi induced by *S. meliloti* Rm41. The data were recorded from ten nodules. Letters indicate different groups analyzed by one-way ANOVA and Tukey post-hoc comparison (alpha=0.05). Error bars are standard error.

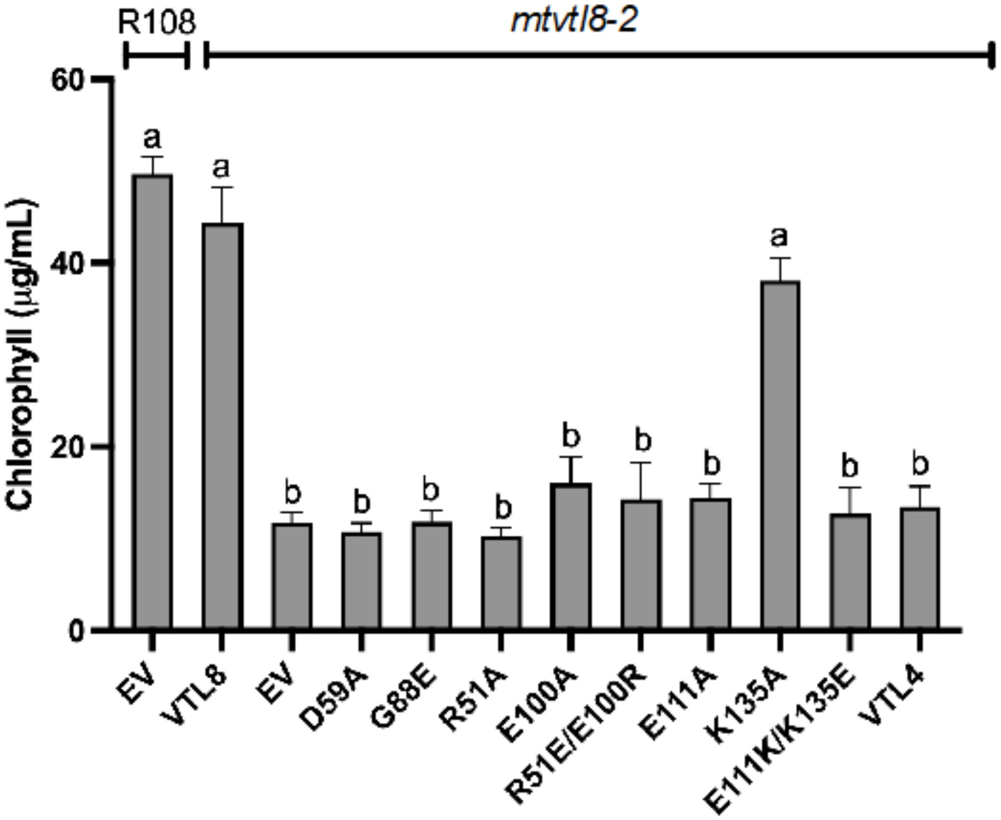


**Supplementary Figure S5. Chlorophyll content in leaves from composite plants in the *in planta* site mutagenesis complementation assessment.**

The chlorophyll content of leaves from R108 and *mtvtl8-2* plants transformed with empty vector (EV), vector expressing *MtVTL8*, different indicated mutants of *MtVTL8*, or *MtVTL4*. N=3. Letters indicate the significant differences analyzed by one-way ANOVA and the Tukey test ($P < 0.0001$). Error bars indicate standard deviation.

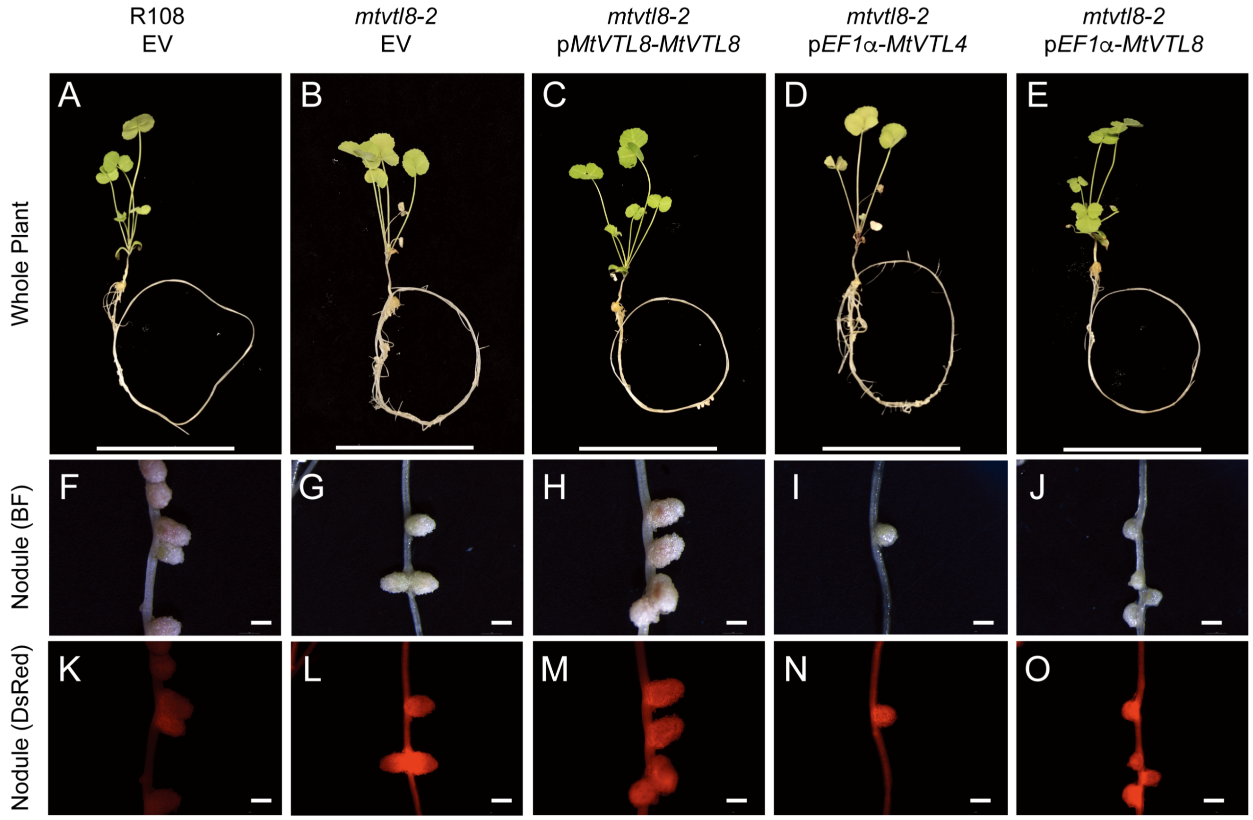


**Supplementary Figure S6.** ***In planta*** **expression of** ***MtVTL4*** **and** ***MtVTL8*** **driven by the** ***EF1a*** **promoter fails to complement** ***mtvtl8-2*** **composite plants.**
**(A-E)** Images of *Medicago truncatula* plants transformed with different vectors. From left to right, **(A)** R108 plant roots transformed with empty vector (EV). **(B-E)** *Mtvtl8* plant roots transformed with **(B)** EV, **(C)** p*MtVTL8-MtVTL8*, **(D)** p*EF1a-MtVTL4* or **(E)** p*EF1a-MtVTL8*. (**F-J**) Bright field (BF) and (**K-O**) DsRed fluorescence images of nodules expressing different constructs corresponding to plants in (**A-E**). Scale bars represent 5 cm for (**A-E**) and 1 mm for (**F-O**).

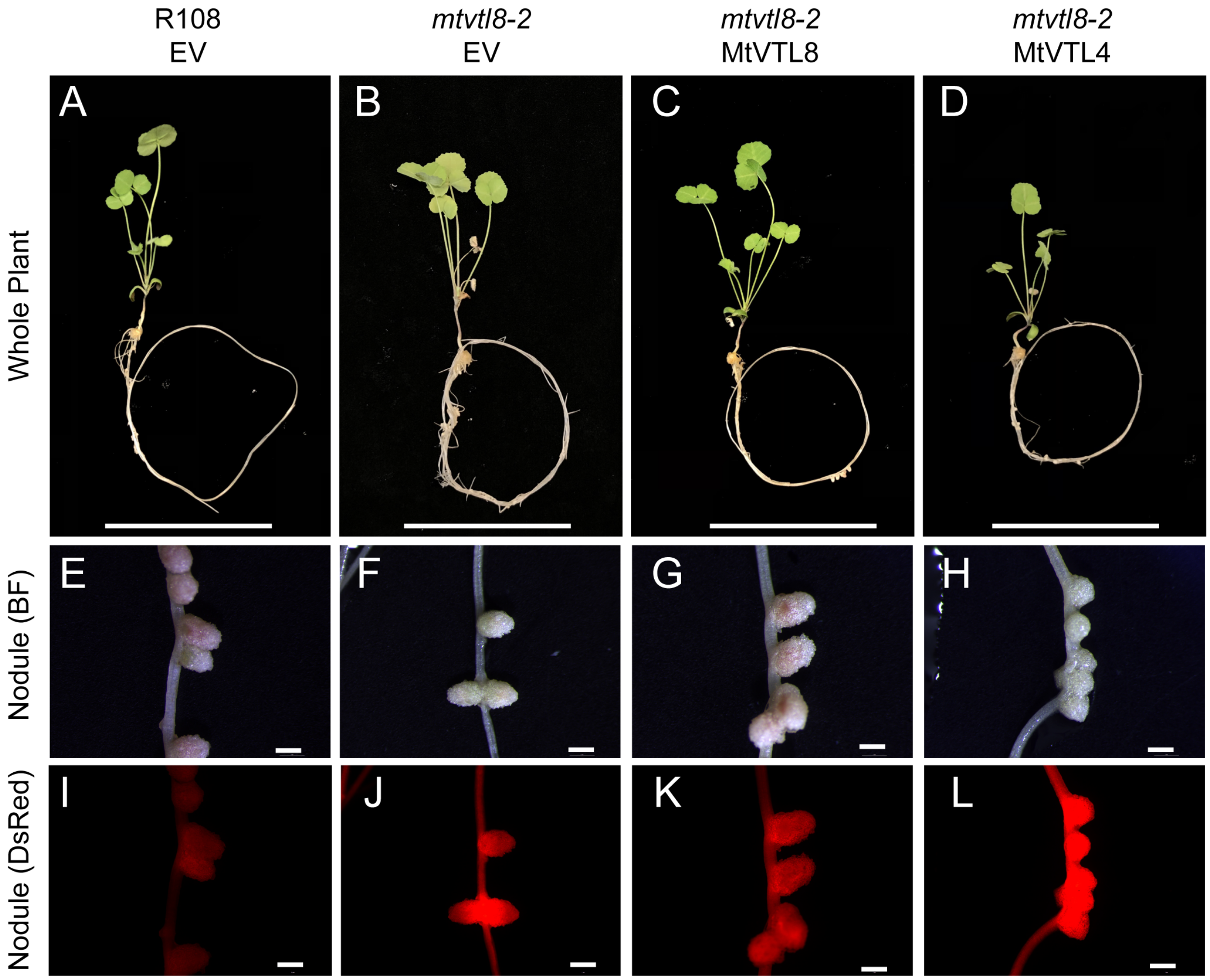


**Supplementary Figure S7. *In planta* expression of *MtVTL4* driven by the *MtVTL8* promoter fails to complement *mtvtl8-2* composite plants.**
**(A-D)** Images of *Medicago truncatula* plants transformed with different vectors. From left to right, **(A)** R108 plant roots transformed with EV; **(B, C, D)** *mtvtl8*-2 plant roots transformed with **(B)** empty vector (EV), **(C)** p*MtVTL8-MtVTL8-MtVTL8t*, **(D)** p*MtVTL8-MtVTL4-MtVTL8t*. **(E-H)** Bright field (BF) and **(I-L)** DsRed fluorescence images of nodules that expressed different constructs corresponding to plants in **(A-D)**. Scale bars represent 5 cm for **(A-D)**. Scale bars represent 1 mm for **(E-L)**.

(A)

```
EgVIT1      --------------------------------MADGANDGGNPGAEEQQRLLDQHKEAHF  28
MtVTL8      ---------------MAVGTICDSESLNKVIT-IPNPTKEMEEKQIKIKENCKTSDIDYW  44

EgVIT1      TAGEIVRDIIIGVSDGLTVPFALAAGLSGANASSSIVLTAGIAEVAAGAISMGLGGYLAA  88
MtVTL8      QRAQWLRAAVLGANDGLVSVASLMMGVGAVKTDSATMLVAGFAGLIAGACGMAIGEFVSV 104

EgVIT1      KSEADNYARELKREQEEIIRVPDTEAAEVAEILARYGIEPHEYGPVVNALRKKPQAWLDF 148
MtVTL8      YTQYEVEIGQMMRDLGTSDRKEKELEIELEKRR--------------------------- 137

EgVIT1      MMKFELGLEKPDPKRALQSAFTIAIAYVLGGLVPLIPYMFIPVARKAVVASVILTLMALL 208
MtVTL8      -------SLPN----PLQAAAASAFSFSIGGLVPLLSGSFIRVYKIRIIAIMAIASLALV 186

EgVIT1      IFGYAKGYFTDNKPFKSALQTALIGAIASAAAFGMAKAVQSENLYFQ--    255
MtVTL8      VFGGVGAMLGKTPKVKSSIRFLLGGWMAMAITFGLTKLLAHCSGLDLEI    235
```

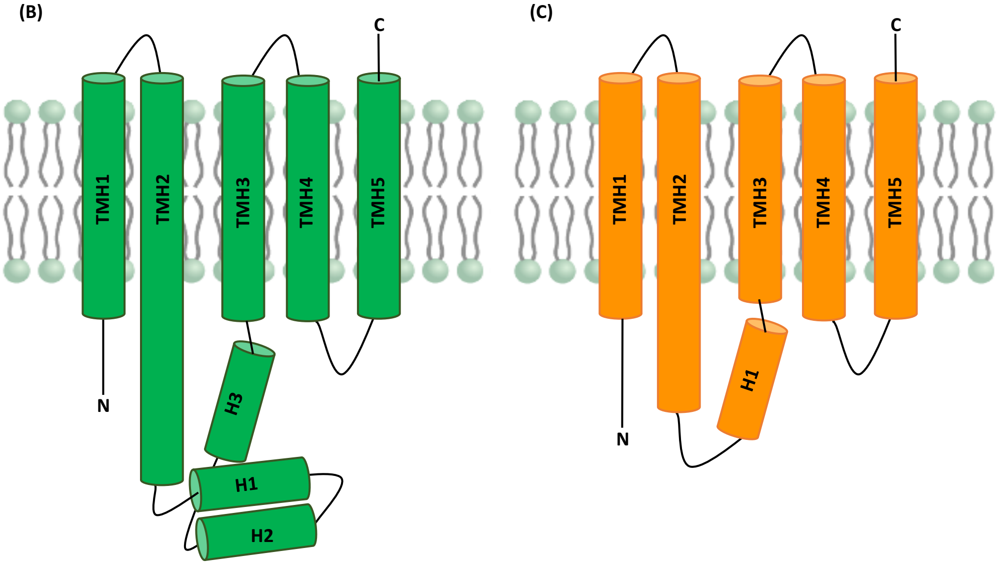


**Supplementary Figure S8. Sequence alignment and topology of EgVIT1 and MtVTL8. (A)** Aligned sequences of EgVIT1 and MtVTL8. Residues in EgVIT1 that were mutated in Kato et al., 2019 and residues in MtVTL8 that were mutated in this work are in bold. Alpha-helices are highlighted in green for EgVIT1 and in orange for MtVTL8. Red arrowheads indicate residues involved in iron binding or transport. Glu72 in EgVIT1 is indicated with a blue arrowhead while the corresponding residue in MtVTL8, Gly88, is indicated by a black/red arrowhead. Orange and green arrowheads indicate residues forming putative salt bridges in MtVTL8. (**B** and **C**) Topology for (**B**) the crystal structure of EgVIT1 and (**C**) the AlphaFold model of MtVTL8. The main difference between the two proteins is the absence of a MBD in MtVTL8 due to a shorter TMH2 and a shorter sequence between TMH2 and 3.

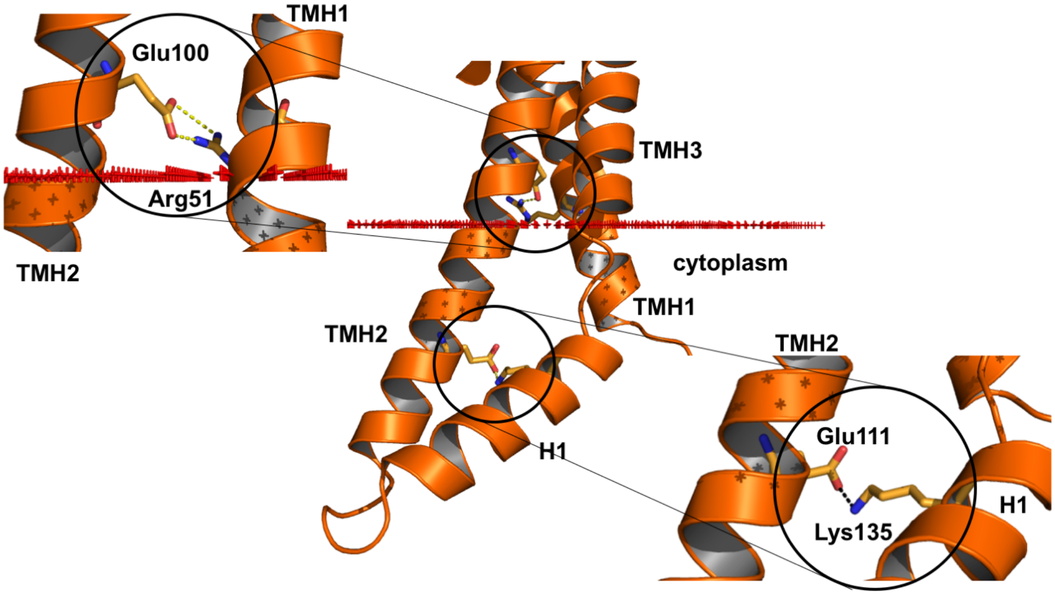


**Supplementary Figure S9. Putative salt bridges in the AlphaFold model of MtVTL8.** Analysis of the AlphaFold model of MtVTL8 revealed two potential salt bridges, one located in the TMD between residues Arg51(TMH1) and Glu100 (TMH2), and the second one in the cytoplasmic region between residues Glu111 (TMH2) and Lys135 (H1). These salt bridges were not observed in the crystal structure of EgVIT1.

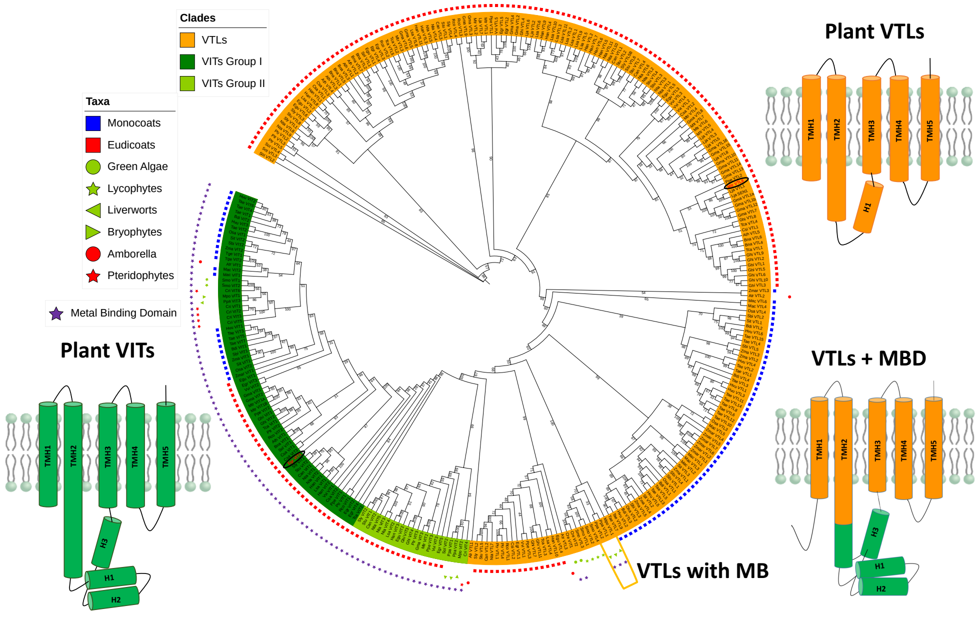


**Supplementary Figure S10. Maximum-likelihood phylogenetic tree of VITs and VTLs.** A maximum likelihood phylogenetic tree was obtained using sequences plant VITs (93 sequences) and VTLs (213 sequences) from 37 plant genomes. Also included are two sequences from an alga. Bootstrap values, in percentages, are shown for each node. Plant VITs (green) are clustered separately from plant VTLs (orange). Plant VITs contain both a TMD and MBD (see cartoon on the bottom left with α-helices in green) while VTLs contain a TMD and one cytoplasmic α-helix (see cartoon on the top right with α-helices in orange). Three VTLs (orange parenthesis) are predicted to contain a MBD similar to VITs (see cartoon on the bottom right with α-helices in orange for the TMD and green for the putative MBD). Some VTLs that lack structural features are clustered in a small subclade (VITs Group II, light green). Abbreviations: Ath (Arabidopsis *thaliana*); Atr (*Amborella trichopoda*); Bdi (*Brachypodium distachyon*); Bna (*Brassica napus*); Can (*Capsicum annuum*); Cre (*Chlamydomonas reinhardtii*); Cri (*Ceratopteris richardii*); Csa (*Cucumis sativus*); Csi (*Citrus sinensis*); Egr (*Eucalyptus grandis*); Egu (*Erythrante guttata*); Ghi (*Gossypium hirsutum*); Gma (*Glycine max*); Han (*Helianthus annuus*); Hvu (*Hordeum vulgare*); Lsa (*Lactuca sativa*); Lja (*Lotus japonicas*); Mac (*Musa acuminata*); Mpo (*Marchantia polymorpha*); Mtr (*Medicago truncatula*); Nta (*Nicotiana tabacum*); Osa (*Oryza sativa*); Ppa (*Physcomitrella patens*); Ptr (*Populus trichocarpa*); Ppe (*Prunus persica*); Rco (*Ricinus communis*); Sbi (*Sorghum bicolor*); Sit (*Setaria italica*); Sly (*Solanum lycopersicum*); Smo (*Selaginella moellendorffii*); Sol (*Spinacia oleracea*); Stu (*Solanum tuberosum*); Tae (*Triticum aestivum*); Tca (*Theobroma cacao*); Tge (*Tulipa gesneriana*); Vvi (*Vitis vinifera*); Zma (*Zea mays*); Zmar (*Zostera* marina). MtVTL8 and EgVIT1 are circled. Taxa are labeled as indicated in the legend. Proteins containing a MBD are labeled with a purple star. The tree is publicly shared on iTOL at this link: https://itol.embl.de/tree/47187131203292916985088806.

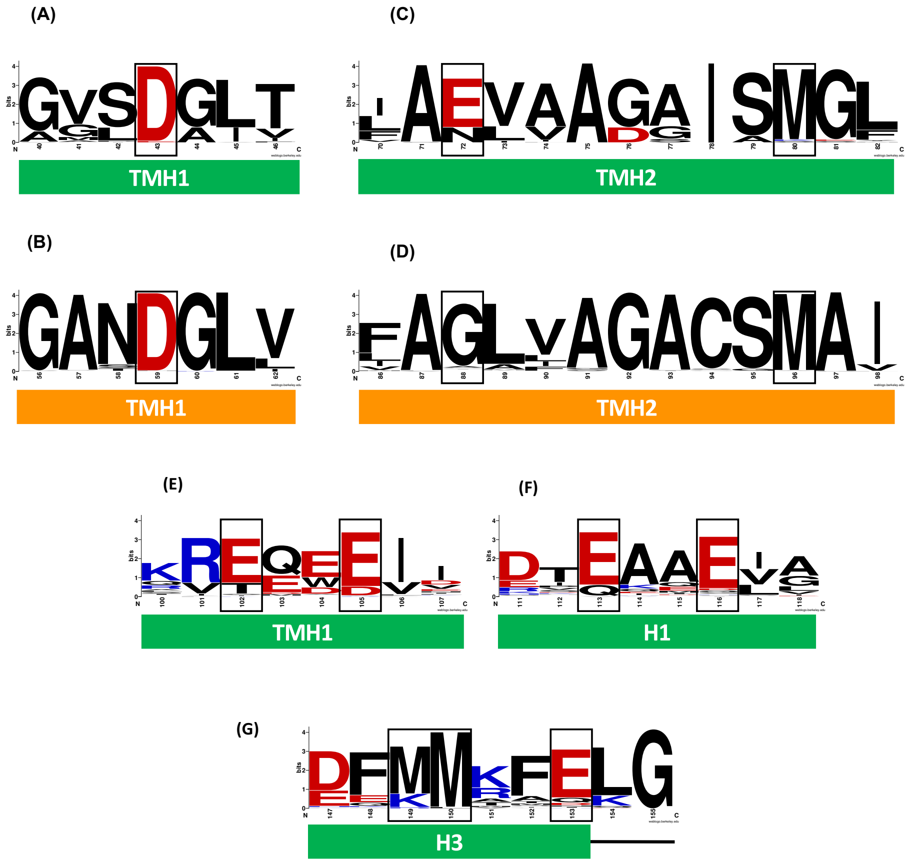


**Supplementary Figure S11. LOGOs of conservation of VIT and VTL residues involved in metal binding and transport. (A-D)** LOGOs of conservation for residues involved in metal binding within the TMD: **(A)** Asp43 on TMH1, **(B)** Asp72 and Met80 on TMH2 in VIT proteins; **(C)** Asp59 on TMH1, **(D)** Gly88 and Met96 on TMH2 in VTL proteins. **(E-G)** LOGOs of conservation for residues involved in metal binding within the cytoplasmic MBD (only VITs): **(E)** C-terminal part of TMH2 and **(F)** N-terminal part of H1 showing the conservation of metal binding residues corresponding to EgVIT1 residues Glu102 and Glu105 on TMH2, Glu113 and Glu116 on H1. **(G)** LOGO of part of H3 from VITs showing the conservation of residues involved in metal binding corresponding to residues Met149, Met150, and Glu153 in EgVIT1. LOGOS were obtained using the aligned sequences of 76 plant VITs or 182 plant VTLs. Green and orange bars represent α-helices for VITs and VTLs, respectively. Numbering is based on the EgVIT1 sequence for VITs and the MtVTL8 sequence for VTLs.

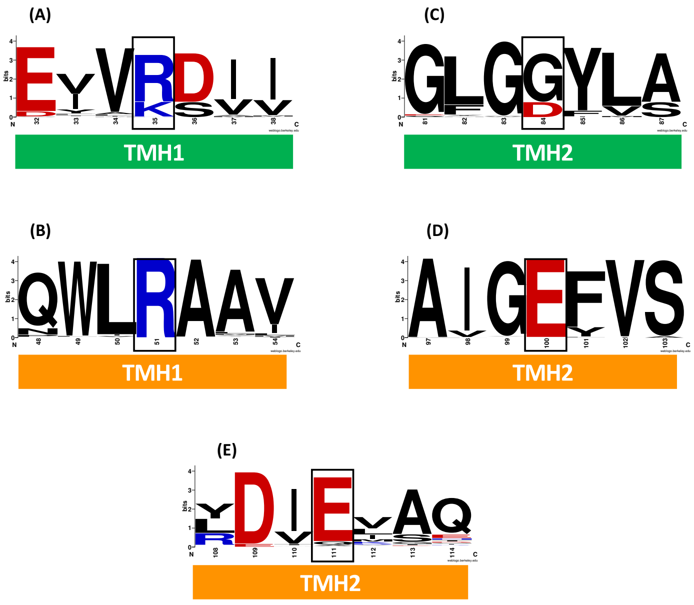


**Supplementary Figure S12. LOGOs of conservation of residues predicted to form two salt bridges in the MtVTL8's AlphaFold model**. **(A-D)** Conservation of residues **(A)** Arg35 and **(C)** Gly84 in VITs, and **(B)** Arg51 and **(D)** Glu100 in VTLs. Arg51 and Glu100 are predicted to form a salt bridge in the MtVTL8 AlphaFold model. Such bridge cannot form in Group I VITs as they lack a negatively charged amino-acid in TMH2. However, Group II VITs have a glutamic acid in this position and could form the salt bridge with the arginine in TMH1. (**E**) Conservation of Glu111 (TMH2) in VTLs. This residue forms a salt bridge with Lys135 (H1). We could not create a LOGO of H1 due to the poor sequence alignment for VTLs in this region. Green and orange bars represent α-helices for VITs and VTLs, respectively. Numbering is based on the EgVIT1 sequence for VITs and the MtVTL8 sequence for VTLs.
